# Realization of frequency hopping characteristics of an epsilon negative metamaterial with high effective medium ratio for multiband microwave applications

**DOI:** 10.1038/s41598-021-96228-4

**Published:** 2021-08-19

**Authors:** Mohammad Tariqul Islam, Md. Moniruzzaman, Touhidul Alam, Md Samsuzzaman, Qutaiba A. Razouqi, Ali F. Almutairi

**Affiliations:** 1 Department of Electrical, Electronic and Systems Engineering, Faculty of Engineering and Built Environment, Universiti Kebangsaan Malaysia, 43600 Bangi, Selangor Malaysia; 2 Space Science Centre, Institute of Climate Change, Universiti Kebangsaan Malaysia, 43600 Bangi, Selangor Malaysia; 3 Department of Computer and Communication Engineering, Faculty of Computer Science and Engineering, Patuakhali Science and Technology University, Dhaka, Bangladesh; 4 Electrical Engineering Department, Kuwait University, 13060 Kuwait City, Kuwait

**Keywords:** Metamaterials, Electrical and electronic engineering

## Abstract

In this paper, a meander-lines-based epsilon negative (ENG) metamaterial (MTM) with a high effective medium ratio (EMR) and near-zero refractive index (NZI) is designed and investigated for multiband microwave applications. The metamaterial unit cell is a modification of the conventional square split-ring resonator in which the meander line concept is utilized. The meander line helps to increase the electrical length of the rings and provides strong multiple resonances within a small dimension. The unit cell of proposed MTM is initiated on a low-cost FR4 substrate of 1.5 mm thick and electrical dimension of 0.06λ × 0.06λ, where wavelength, λ is calculated at the lowest resonance frequency (2.48 GHz). The MTM provides four major resonances of transmission coefficient (S_21_) at 2.48, 4.28, 9.36, and 13.7 GHz covering S, C, X, and Ku bands. It shows negative permittivity, near-zero permeability, and near-zero refractive index in the vicinity of these resonances. The equivalent circuit is designed and modeled in Advanced Design System (ADS) software. The simulated S_21_ of the MTM unit cell is compared with the measured one and both show close similarity. The array performance of the MTM is also evaluated by using 2 × 2, 4 × 4, and 8 × 8 arrays that show close resemblance with the unit cell. The MTM offers a high effective medium ratio (EMR) of 15.1, indicating the design's compactness. The frequency hopping characteristics of the proposed MTM is investigated by open and short-circuited the three outer rings split gaps by using three switches. Eight different combinations of the switching states provide eight different sets of multiband resonances within 2–18 GHz; those give the flexibility of using the proposed MTM operating in various frequency bands. For its small dimension, NZI, high EMR, and frequency hopping characteristics through switching, this metamaterial can be utilized for multiband microwave applications, especially to enhance the gain of multiband antennas.

## Introduction

Metamaterials are getting significant attention in the research community for the last few decades due to their unique electromagnetic characteristics. The metamaterial structures usually belong to modern physics and play an essential role in scattering the incoming waves in a particular direction. The geometry of the radiation structure generally depending upon the operating frequency band. The electromagnetic-based metamaterials are a new class of composite materials with extraordinary electromagnetic characteristics of negative permeability, negative permittivity, negative refractive index. These characteristics are not typically observed in the existing natural materials. A Russian physicist Victor Veselago provided a new theoretical explanation of a material that is different from present materials in nature. These materials are usually a periodic arrangement of sub-wavelength elements. The unique electromagnetic characteristic in metamaterials can be attained by employing various geometrical structures instead of chemical accounts. In electromagnetic and microwave applications, materials are chosen based on dielectric properties. The metamaterials applications include but are not limited to: mutual coupling suppression in MIMO elements^1^, RF shielding^2^, Antenna Radar Cross Section (RCS) reduction^3^, antenna gain/directivity enhancement^4^, invisibility cloaking^5^, and miniaturized sophisticated structures for the RF sensing applications^6^.

Different metamaterial structures are reported in the existing literature to achieve negative permeability, negative permittivity by employing different design configurations. In^7^, a miniaturized metamaterial structure with dimensions 5.5 × 5.5 mm and an EMR of 8 was proposed for C-band microwave applications. The design configuration was realized on a 1.57 mm thick low-loss Rogers 5880 substrate to achieve the double negative metamaterial characteristic in the intended frequency band. In^8^, a uniplanar left-handed metamaterial was reported for the terrestrial microwave links. The coupled split-rings were employed on the top-layer to achieve the multi-band response in the C-band (4–8 GHz) applications. The proposed design has a large size of 30 mm × 18 mm with an EMR value of 4.5. Moreover, this type of metamaterial configuration can be used for civil and navy organizations for weather forecasting to achieve uninterrupted connectivity. In^9^, A dual-band metamaterial design was proposed for the 8 to 16 GHz frequency band. The single negative metamaterial (μ < 0) characteristics were achieved by introducing multiple modified splits in the square ring and metallic strip at the rear side. In another study, a miniaturized metamaterial-based with split-ring resonators was proposed in^10^ to isolate the array elements in X-band applications. In^11^, a multi-band epsilon negative metamaterial with a high effective medium ratio was proposed for the S, C, and X-band applications. The metamaterial effective characteristics were achieved by employing the coupled split-ring resonators on the top-layer. This design configuration has a compact size of 8 × 8 mm^2^ on a low-cost FR-4 substrate. Moreover, a high EMR value of 16.74 was achieved for the single negative metamaterial. A metasurface is presented by Zhang, K et al*.* that generates distinct orbital angular momentum modes to be engaged in cross and copolarized output channels^12^. In another paper, a metasurface is constructed that exhibits phase modulation helping to attain co and crosspolariazation fields as output when circularly polarized incident wave is intruduced^13^. Moreover, polarization manipulation is performed by using meta-detectors that provides a freedom of electromagnetic waves within a larger bandwidth^14^. In another study, epsilon negative metamaterial integrated with cross-coupled ring resonator for multiband satellite and radar communications is proposed in^15^. A high surface current concentration along with high E and H-field was observed on the coupled split ring. This design configuration provides EMR value of 8.03 along with dimensions of 9 × 9 mm^2^. In^16^, MTM unit cell of Jerusalem cross-shaped is designed that shows NZI property that has been used to increase the patch antenna gain operating at 43 GHz. A split-ring resonator(SRR) based symmetrical metamaterial is reported in^17^ that reveals ENG property and can be utilized to reduce the mutual coupling effect between two antennas operating in the S-band. In another paper presented by T. Shabbir et al*.* uses double-ring diagonal coupled SRR MTM to provide decoupling effect in 16 port MIMO antennas^18^. An annular slot resonator base MTM is presented in^19^ that is used with an antenna to obtain high gain. Similarly, several metamaterials are also reported in^20–22^ which are used for performance increasing in antennas. An MTM based sensor in which a single circular SRR is used for fluid and strain sensing purposes^23^. Whereas, in^24^ metamaterial absorber is used for grain sense. In another literature, the MTM emitter is presented which is used for gas sensing purposes^25^. An MTM based on an octagonal-shaped resonator is reported in^26^, which utilizes the broadband absorber property of MTM to harvest energy from the WiMAX band. On the other hand, split ring arrays are presented by N Misran et al*.* where parameterization is accomplished based on substrate thickness and permittivity^27^.

As the metamaterial finds its utility in various applications as discussed above, in this manuscript, a meander lines-based multiband epsilon negative metamaterial is designed to cover the S, C, X, and Ku-bands with frequency. The proposed metamaterial structure is designed on a low-cost FR-4 substrate with a thickness of 1.5 mm. The resonating array elements are employed at the top-layer, whereas the bottom layer is left a void. A commercially available 3D electromagnetic Computer Simulation Technology (CST) software is used to design and analyze the meander lines-based metamaterial design. A popularly used Nicolson-Ross-Wier (NRW) method is utilized to extract the metamaterial effective parameters (permittivity, permeability, and refractive index). The performance of the proposed structure is also analyzed in terms of surface current distribution, electric field, and magnetic field distribution. An equivalent circuit modeling is performed, and lumped components values are extracted by using the Advanced Design System Software (ADS). An effective medium ratio (EMR) is calculated, which shows the compactness of the design with an EMR of 15. Additionally, different array configurations (1 × 2, 2 × 2, and 8 × 8) are also analyzed. The frequency hopping characteristics are investigated through numerical simulations by open and short circuited of the split gaps of the three outer rings using three switches at these positons. This manuscript is managed as follows: in Section two, the design and analysis of metamaterial are discussed. Parametric studies on the proposed MTM is made at section three based on split gaps, substrate materials and substrate thickness. The theory on MTM and its property extraction is discussed in section four whereas the electric field, surface current, and magnetic field distribution are discussed in Section five. Section six is intended to equivalent circuit modeling whereas analysis of simulation and measured results is done in section seven that includes analyzing extracted parameters, measurement setup, array performance analysis, numerical analysis on frequency hopping and performance comparison of the proposed MTM with existing literature. Finally, the manuscript is concluded in section eight focusing on the major outcomes of the proposed MTM.

## Metamaterial unit cell design and simulation

**Figure 1:**
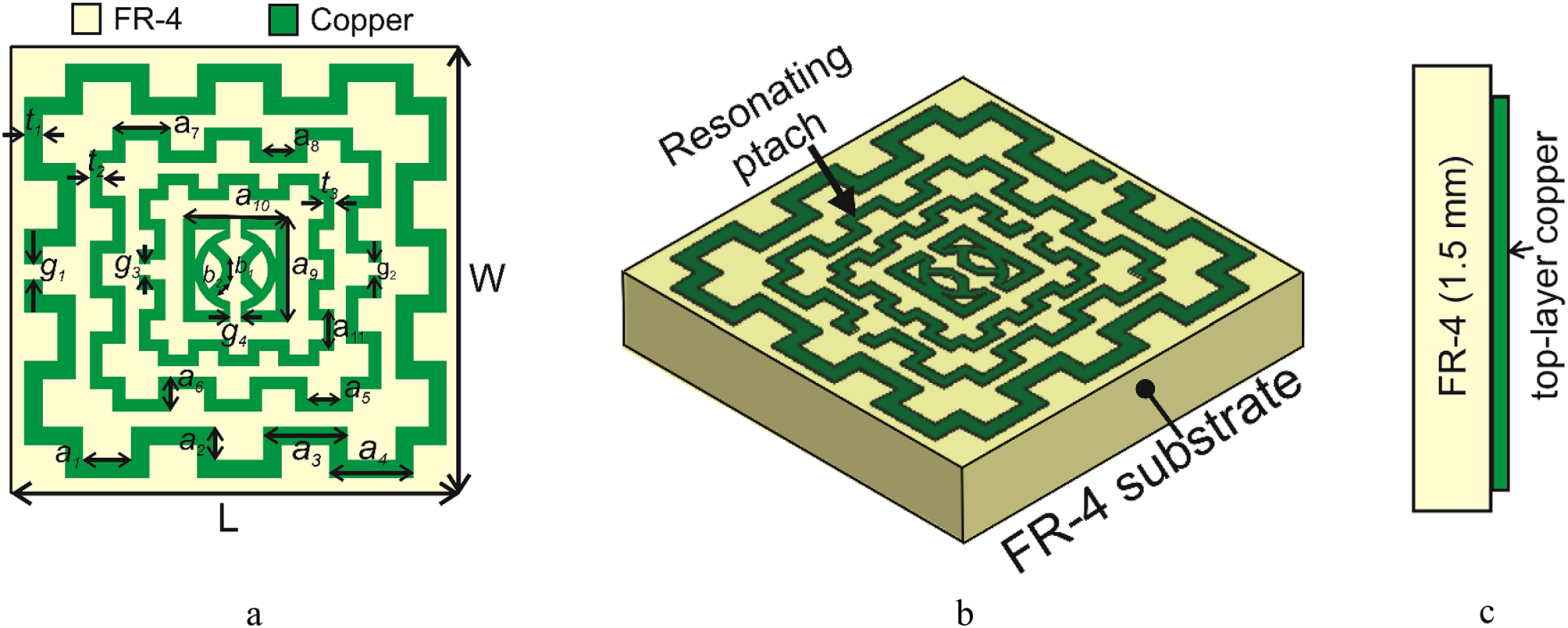
Geometric structure of the proposed MTM unit cell. (CST STUDIO SUITE 2019, https://www.3ds.com/products-services/simulia/products/cst-studio-suite)^28^.

**Figure 2:**
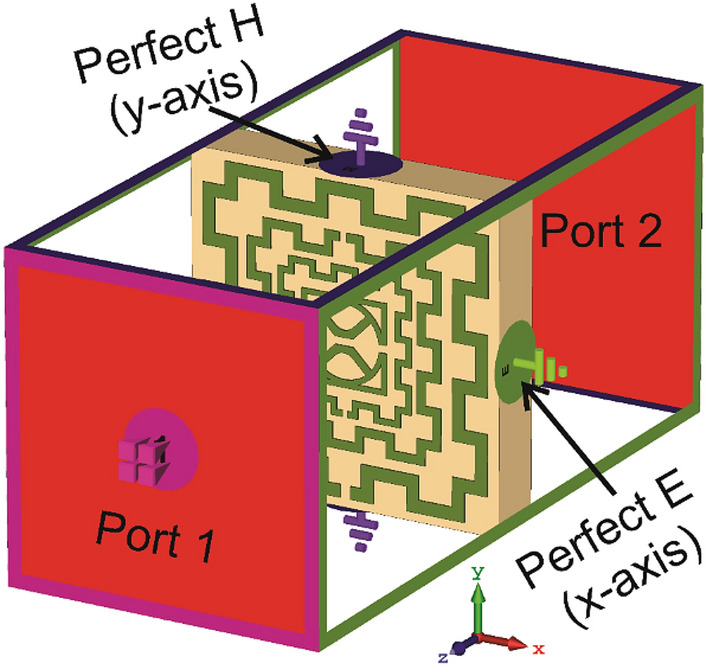
MTM unit simulation setup in CST. (CST STUDIO SUITE 2019, https://www.3ds.com/products-services/simulia/products/cst-studio-suite)^28^.

**Figure 3:**
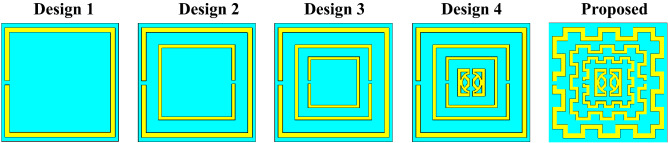
MTM unit cell design methodology. (CST STUDIO SUITE 2019, https://www.3ds.com/products-services/simulia/products/cst-studio-suite)^28^.

**Figure 4:**
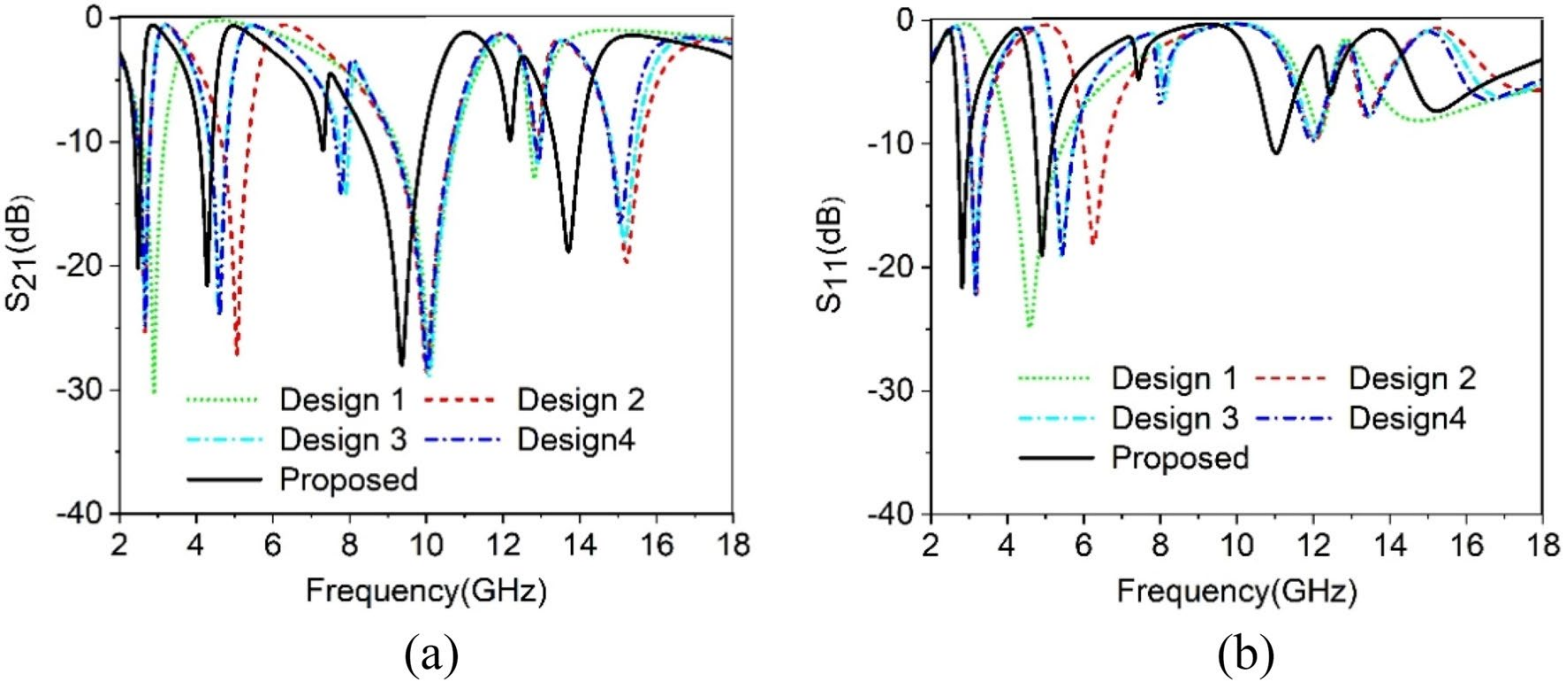
(**a**) Transmission coefficient(|S_21_|) of proposed MTM unit cell (Design methodology). (**b**) Reflection coefficient(|S_11_|) for various changes in design (Design methodology).

**Figure 5:**
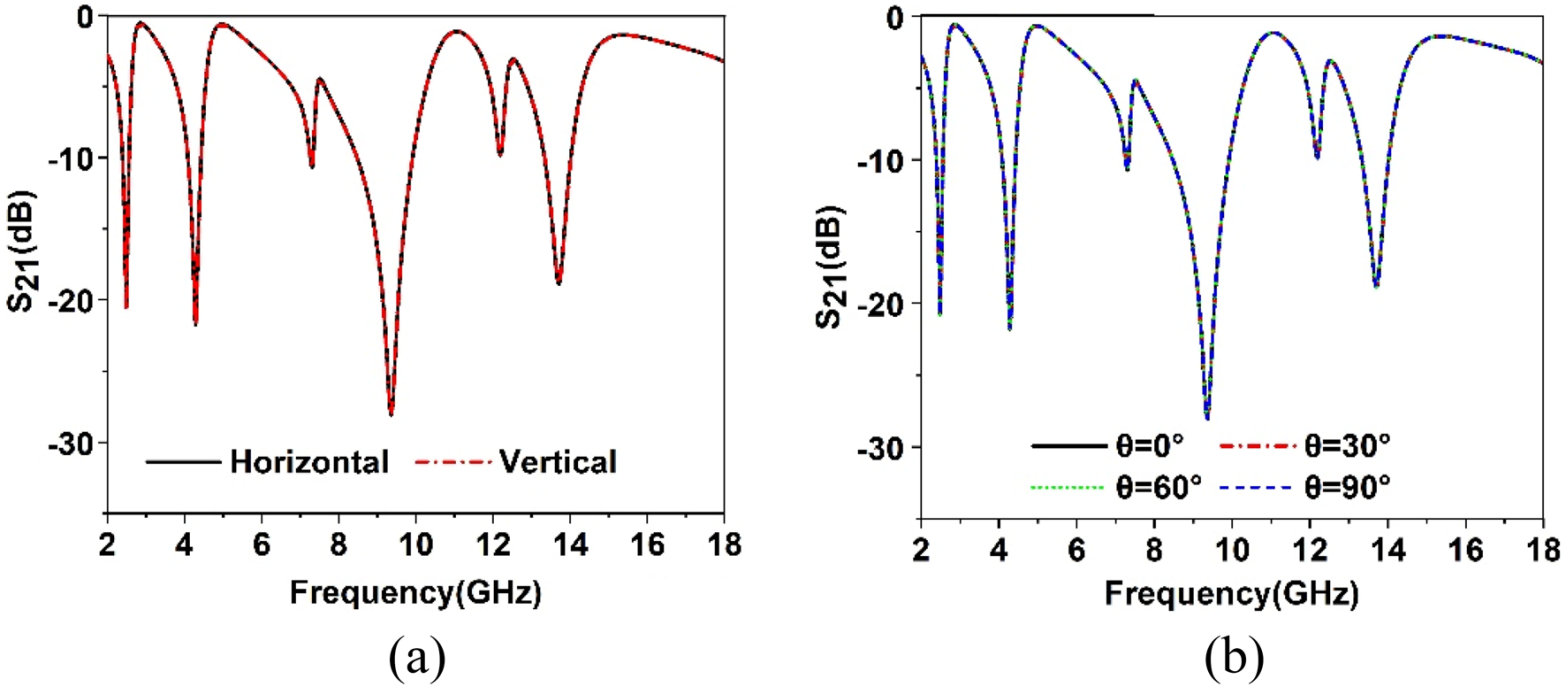
(**a**) S_21_ for horizontal and vertical polarization of the incident wave (**b**) S_21_ response for different oblique incident angle (θ).

The Design of the proposed MTM unit cell is initiated on an FR-4 substrate with a dimension of 8 × 8 mm^2^ and 1.5 mm thickness. The substrate material exhibits a permittivity of 4.4 with a loss tangent of 0.02. The schematic diagram of the unit cell is shown in Fig. 1, in which Fig. 1a presents the front view of the structure, whereas Fig. 1b exhibits the top view and Fig. 1c is the side view. The unit cell consists of three modified square split ring resonators loaded with splitted square ring coupled with modified semicircle shapes. The outer three rings are modified in meander lines patteren. The length and width of the rings and inter ring distances are seleceted by numerous numerical simulations performed in CST microwave studio suite-2019^28^. The simulation setup is exhibited in Fig. 2 where normally incident transverse electromagnetic (TEM) wave is exposed on the MTM unit cell. As expressed in this Figure, waveguide ports are used in the z-axis that is the direction of the incident electromagnetic wave. Two boundary conditions are applied in the x-axis and y-axis where the first axis is employed for the E field boundary and the next one is for the H field boundary. The step by step design method is shown in Fig. 3. The design is initiated with the outer split ring resonator as shown in design 1 of Fig. 3. This split ring is situated at a distance of 0.22 mm from the edge of the substrate with a length of approximately 7.55 mm and width of 0.33 mm. the ring creates resonance when propagating electromagnetic wave incidences on it as the conducting part of the ring exhibits inductive property whereas split gap forms the capacitance. As shown in Fig. 4a, three resonances of transmission coeffiecient (S_21_) are obtained due to the outer ring at frequencies of 2.9 GHz, 10.1 GHz and 12.84 GHz, respectively. Corresponding reflection coefficient (S_11_) is displayed in Fig. 4b that shows one sharp resonance at 4.5 GHz. In design 2 of Fig. 3, second split ring resonator is added which results two additional resonances of S_21_ at 5 GHz and 15.2 GHz. The addition of this resonator not only contributes to the new resonances but also introduce mutual coupling between the two rings. Due to the mutual coupling effect the previous resonance frequencies are not only shifted towards the lower frequencies but also their magnitudes are changed.The corresponding changes of S_11_ are also shown in Fig. 4b that shows S_11_ resonances around 3.16, 6.24, 12.1, and 13.4 GHz. In design-3 of Fig. 3, another ring is included whose split gap is oppositely positioned compared to the second ring.The effect of this inclusion is seen in Fig. 4, with a right shift of all the resonances of S_21_ with an addition resonance at 7.89 GHz. The corresponding reflection coefficient is also presented in Fig. 4b. Now, design 4 is obtained by inserting a square split ring coupled with a modified semi-circle. The inclusion of this part causes electromagnetic interaction with the field obtained from earlier rings and thus shifted the resonances towards the lower values. In the final steps the outer three rings are modified with meander lines pattern that increases the elecetical length of the outer rings. Due to this increased conducting length inductive effect is more pronounced that causes a tremendous shifts in resonance frequencies towards the lower values as shown in Fig. 4. This final design provides four major resonances at 2.48, 4.28, 9.36, 13.7 GHz covering S, C, X, and Ku bands. The various dimensions of the proposed unit cell is shown in Table 1. The results obtained in Fig. 4a,b is due to vertical polarization of the incident wave. The effect of the change of the polarization angle is studied further for two different esteem of polarizations. Figure 5a shows the S_21_ comparison for the vertical and horizontal polarization of the incident wave indicating that in both cases proposed MTM exhibits the similar response. The S_21_ response also observed for different angle of incident wave that is depicted in Fig. 5b. The effect of this oblique incidence is studied for varying the incident angle, θ from 0° to 90° with four equal steps. It is noticed that transmission coefficient is unaffected due to the change of the incident angle indicating that proposed MTM structure shows similar resoponse for any angle of incidence. Thus, the proposed MTM is insensitive to the variation of oblique incindent angle.

**Table 1:** Specifications of the various parameters of the proposed MTM unit cell.

Parameter	Dimension	Parameter	Dimension	Parameter	Dimension	Parameter	Dimension
W	8	L	8	a1	0.86	a2	0.6
a3	1.5	a4	1.5	a5	0.6	a6	0.6
a7	1.75	a8	0.6	a9	1.9	a10	1.9
a11	0.7	b1	0.5	b2	0.34	t1	0.33
t2	0.2	t3	0.2	g1	0.27	g2	0.23
g3	0.2	g4	0.2

## Parametric study on the proposed MTM unit cell

The substrate material, its thickness, the dimension of copper rings, split gaps are some key factors for the performance of the metamaterials. Due to these factors, inductance and capacitance are modified, which causes a change in the resonance frequencies of the metamaterial. In this section, parametric studies are performed to investigate the effects of modification of split gaps, substrate thickness, and substrate materials.

### Effect of changing the split gaps

**Figure 6:**
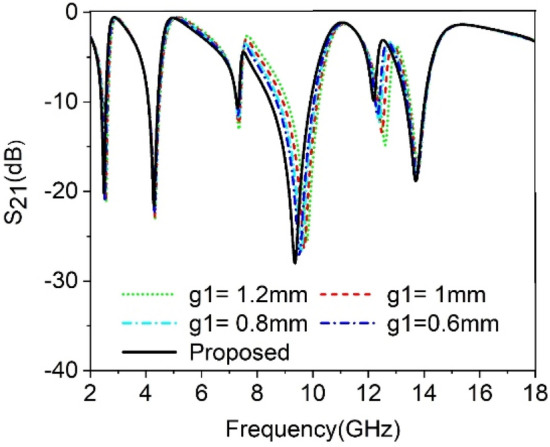
The transmission coefficient of the proposed unit cell for split gap variation, *g1.*

**Figure 7:**
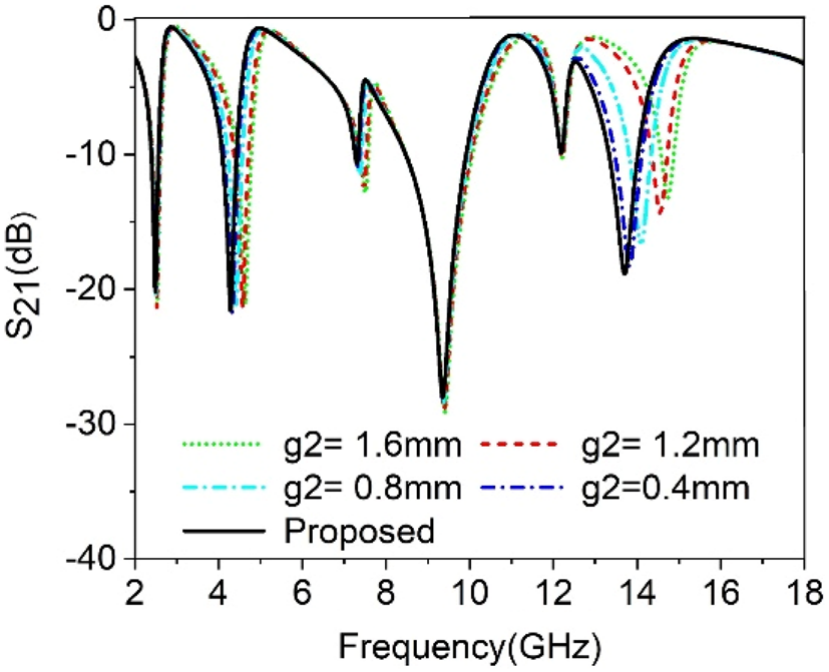
The transmission coefficient of the proposed unit cell for split gap variation, *g2.*

**Figure 8:**
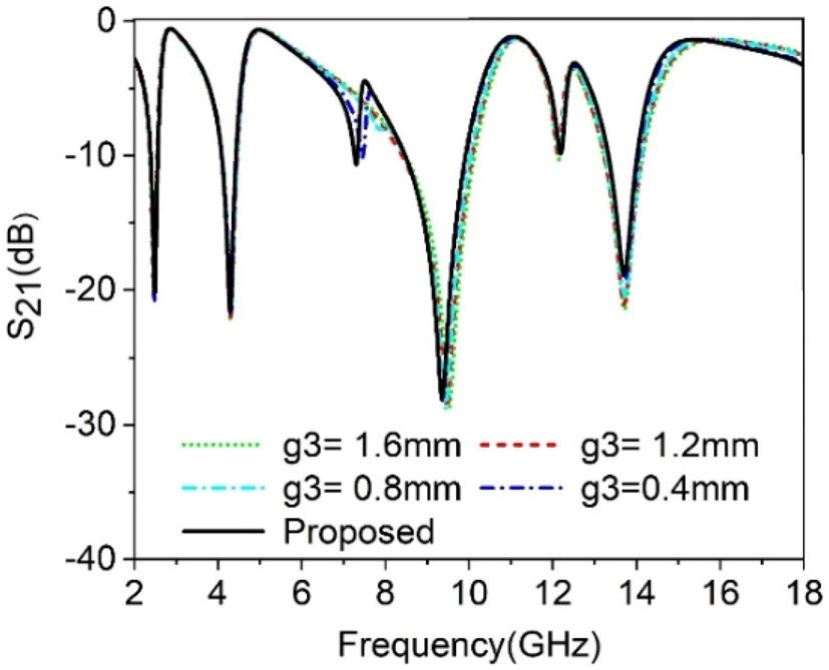
The transmission coefficient of the proposed unit cell for split gap variation, *g3.*

The split gaps that existed in various rings have the dominant effect to control the response of the proposed MTM unit cell. As split gap in each ring of the proposed MTM causes to create capacitance, any change of the capacitance value affects the resonance of the MTM due to the inherent relation between capacitance and resonance frequency. In this study, the split gap *g1*, *g2* and *g3* of the of three outer rings are changed one at a time, keeping others constant and the effect of this is observed. Figure 6 depicts the outcome for the change of split gap *g1* of the outermost ring. From this figure it is realized that any change of the g1 has a dominant effect on the resonance occurred at 9.36 GHz. As *g1* decreases from 1.2 mm to 0.27 mm, the capacitance due to split gap,*g1* increases gradually. Thus it reduces the resonance frequency towards the lower values since resonance frequency, $= \frac{1}{2\pi \sqrt{LC}}$. The impact of the change in second ring split gap, *g2* is displayed in Fig. 7. The modification of capacitance values due to the change in *g2* causes a changing effect at all of the resonance frequencies, with a more pronounced effect observed around the resonances within 4.3 GHz and 13.7 GHz. The variation of split gap, *g3* of the third ring shows its impact on the resonance occurred at 9.36 GHz as shown in Fig. 8 though the variation of the resonance frequency is less compared to the effect of change of the *g1*. It is observed that a decreasing value of *g3* contributes significantly to minor resonance existing around 7.3 GHz. Thus split gap distance plays a vital role in modulating the capacitance of the resonator and in this way, the resonance frequency can be modulated by adjusting the gap distance.

### Effect of substrate material on the performance of MTM

**Figure 9:**
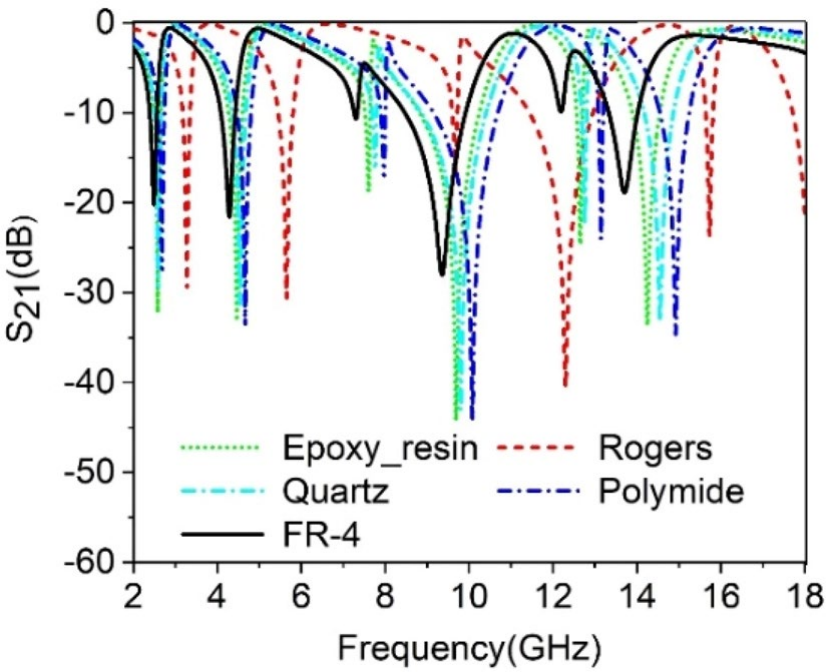
Study the effect of different substrate materials on S_21_.

The effect of various substrate materials has been studied to understand the behavior of the proposed MTM. Five different substrate materials such as Epoxy-resin, Rogers, Quartz, Polymide, and FR-4 are selected for studying the effect. These materials are varied in their properties, such as permittivity, loss tangent, and thermal conductivity. The materials are considered to have the same thickness and permeability constant. The variation of S_21_ for different substrate materials is shown in Fig. 9. FR-4 inherits the highest permittivity of 4.4 with a loss tangent of 0.02. In comparison, Rogers has the lowest permittivity of 2 with a loss tangent of 0.0021. Epoxy-resin, Quartz, and Polymide show the permittivity of 4, 3.75, and 3.5, respectively, with loss tangent values of 0.0, 0.0004, and 0.0, respectively. FR-4 is a flame-resistant composite material composed of fiberglass with epoxy resin that shows resonances at lower frequencies than the other materials. On the other hand, Rogers(RT5880) are composed of glass microfiber reinforce PTFE with a low dissipation factor makes it suitable for high-frequency applications. The S_21_ response shifts towards the high frequency for Rogers substrate, as shown in Fig. 9. In the case of Quartz, Epoxy-resin, Polymide, the resonance frequency varies with one another, which indicates that the performance of the MTM depends on the substrate material. A correlation can be made with the permittivity of the substrate material because the high permittivity causes to increase in the capacitance of the MTM; thus, the resonance frequency shifts towards the lower value. Thus, FR-4, due to its highest permittivity within the examined material, shows the lowest resonance frequency. As the material's permittivity decreases, the resonance frequency shifts towards the higher frequency in Quartz, Polymide, Epoxy-resin, and Rogers. From Fig. 9, it is also noticed that very sharp resonances are detected in the case of materials such as Epoxy-resin, Quartz, and Polymide due to their very low loss tangent. Within these materials, Rogers is a more attractive material due to its capability to operate in worse environments, but it is a more costly PCB material than others. On the other hand, FR-4 is more popular due to its high mechanical strength, thermal stability, and flame redundancy level, making it suitable for most electrical and mechanical performance requirements.

### Effect of the substrate thickness on MTM performance

**Figure 10:**
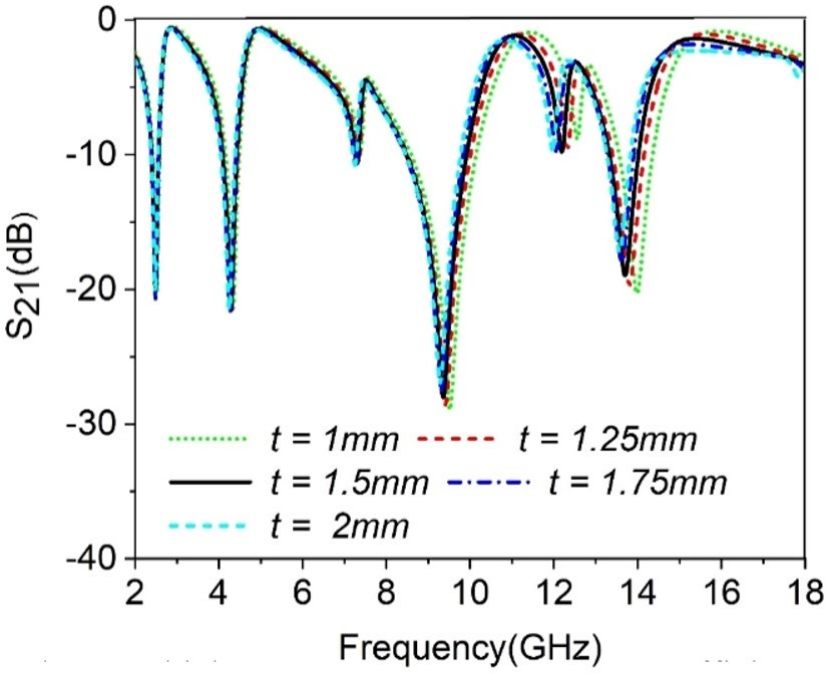
Effect of substrate thickness, *t* on the transmission coefficient of the Proposed MTM.

A study on the thickness of the substrate material is accomplished to realize it’s effect on the S_21_ performance. The Substrate thickness has been increased from 1 to 2 mm with an equal distance of 0.25 mm. From Fig. 10, it is observed that substrate thickness shows an impact on the resonance frequencies though the resonances that occurred in the low frequencies are less affected due to the change of it. But as the frequency increases, the shift of the resonance frequencies due to the change of the substrate thickness is more observable as the substrate acts as the dielectric medium between the two waveguide ports. The dielectric effect of the substrate is a factor of substrate thickness. Moreover, the resonator's electric field is not solely concentrated on the resonator part; instead, it extends within the substrate material. In the case of the substrate's small thickness, this fringing field contributes to the strip capacitance formed by the SRR. As the substrate thickness increases, the fringing electric field affected in substrate becomes less pronounced, and parallel capacitance increases due to this change. Eventually, this parallel capacitance shows an impact in high-frequency resonances, and as the thickness increases, resonance frequency shifts towards the lower values.

## Theory on metamaterial property extraction method

The researchers have investigated various models to realized the frequency-dependent property of the metamaterial. Within those, the Lorentz model is popularly used for metamaterial characterization. In the Lorentz model, electrons' movement in metamaterial due to the excitation of an electric field can be presented as a damped harmonic oscillation consisting of the polarization field. Mathematically the relation between the electric field and polarization field can be explained by using Eq. (1)^29^:

1 $$\frac{{d^{2} }}{{dt^{2} }}P_{i} + {\Gamma }_{L} \frac{d}{dt}P_{i} + \omega_{0}^{2} P_{i} = \varepsilon_{0} \chi_{L} E_{i}$$

In Eq. (1), acceleration of charges is presented by the first term of the left side, whereas damping is presented by the second term having damping coefficient, ${\Gamma }_{L}$ and restoring force is represented by the third term. The excitation field or driving field is placed at the right side of Eq. (1), where the coupling coefficient, ${\chi }_{L}$ is also incorporated. One solution of the Eq. (1) provides electric susceptibility in terms of polarization field and excitation field and can be presented as Eq. (2)^29^:

2 $$\chi_{e, Lorentz} \left( \omega \right) = \frac{{P_{i} \left( \omega \right)}}{{\varepsilon_{0} E_{i} \left( \omega \right)}} = \frac{{\chi_{L} }}{{ - \omega^{2} + j{\Gamma }_{L} \omega + \omega_{0}^{2} }}$$

The electric permittivity of the Lorentz model can be defined in terms of Eq. (2) and it takes the form of Eq. (3).

3 $$\varepsilon_{Lorentz} \left( \omega \right) = \varepsilon_{0} \left[ {1 + \chi_{e, Lorentz} \left( \omega \right)} \right]$$

From the Lorentz model of electromagnetic characteristics, the Drude model can be derived by eliminating the restoring force from Eq. (1) and the solution of it provides an expression for electric susceptibility of the Drude model as expressed in the following equation^29^:

4 $$\chi_{e, Drude} \left( \omega \right) = \frac{{\chi_{D} }}{{j{\Gamma }_{D} \omega + \omega_{0}^{2} }}$$

In this model, plasma frequency is considered as an alternative representation of the coupling coefficient with a relation of ${\chi }_{D}={\omega }_{p}^{2}$. In the case of a positive coupling coefficient, the Lorentz model's resonant nature provides a narrow band negative real permittivity at frequencies higher than the resonance frequencies. On the other hand, for $\omega <\sqrt{{\omega }_{p}^{2}-{\Gamma }_{D}^{2}}$ wide spectral negative permittivity can be observed in this model. The metamaterial unit cell's behavior can be replicated in CST microwave studio, which uses Drude-Lorentz variables as the parameter to obtain scattering parameters by the process of numerical simulations^30^. For identifying effective parameters, numerous methods have been reported in the literature. In^25^, homogenization theory is employed in which metamaterial is considered an isotropic homogeneous slab. Thus, microscopic complexity can be handled in a macroscopic view when an electromagnetic wave impinges on an object. The homogenization theory is also applied in robust retrieval^25^ method in which the metamaterial slab's boundaries are carefully determined to ensure constant impedance for varying thickness of slab as in reality; the metamaterial is not a homogeneous medium. The sensitivity of impedance and refractive index for the small variation of S parameters are also addressed in this method as determined S parameters by numerical simulation are noisy. Mathematical form to extract effective permittivity and permeability is also presented in^31^ based on the most popular method of effective parameter extraction named Nicolson-Ross-Wier (NRW)^32,33^ in which transmission coefficient, S_21_ and reflection coefficient, S_11_ are employed to evaulatue permittivity, permeability, refractive index and impedance. For,

5 $$V_{1} = |S_{11} + |S_{21} |$$

6 $$V_{2} = \left| {S_{21} \left| - \right|S_{11} } \right|$$

The expressions for relative permittivity, ε_r_, and relative permeability, µ_r_ take the forms of Eqs. (7) and (8)^31^.

7 $$\varepsilon_{r} \sim \frac{2}{{jk_{0} d}} \times \frac{{\left( {1 - V_{1} } \right)}}{{\left( {1 + V_{1} } \right)}}$$

8 $$\mu_{r} \sim \frac{2}{{jk_{0} d}} \times \frac{{\left( {1 - V_{2} } \right)}}{{\left( {1 + V_{2} } \right)}}$$

where ${k}_{0}=\frac{2\pi f}{c}$, c is the velocity of light, and d is the thickness of the substrate.

The expression for the refractive index, *n*_*r*_ is represented as Eq. (9).

9 $$n_{r} = \sqrt {\varepsilon_{r} \mu_{r} }$$

In the expression for normalized impedance, Z can be directly obtained from the scattering parameters and is written in Eq. (10)^34^.

10 $$Z = \sqrt {\frac{{\left( {1 + S_{11} } \right)^{2} - S_{21}^{2} }}{{\left( {1 - S_{11} } \right)^{2} - S_{21}^{2} }}}$$

MATLAB code based on the Eqs. (7)–(10) has been used to determine effective parameters in association with the data obtained from CST microwave studio regarding S parameters.

## Surface current, electric and magnetic field analysis

When a plane wave incident on a metamaterial surface, a scattering field is created due to the induced current in the metallic part of the metamaterial. The interrelation among the electric field, magnetic field, and surface current in a metamaterial can be well described with Maxwell’s equations. A current through conducting element in a metamaterial produces a magnetic field; on the other hand, changing the magnetic field can induce electromotive force. Thus, current fields of electricity and magnetism are associated with each other through Ampere’s and Faraday’s law four differential equations as described by Maxwell, summarizes these phenomena^35^:

11 $$\nabla .E = \rho_{v} \left( t \right)/\varepsilon$$

12 $$\nabla .{\text{B}} = 0$$

13 $$\nabla \times E = - \mu \partial H/\partial t$$

14 $$\nabla \times B = J\left( t \right) + \varepsilon \partial E/\partial t$$

The electromagnetic behavior in a medium is also controlled by the permittivity, permeability, and conductivity $\sigma$ of the medium. Along with these parameters, the medium's boundary condition also significantly influences the electromagnetic properties. The following two equations provide a summarized representation of the interrelation between electromagnetic fields and material properties.

15 $${\text{D}} = \varepsilon {\text{E}}$$

16 $${\text{B}} = \mu {\text{H}}$$

In these equations, ${\rho }_{v}$ and *J* is the density of charge and surface current, respectively, whereas $\upvarepsilon$, electric permittivity, and $\mu$, the magnetic permeability. Within the other terms, B and D symbolize fluxes of the magnetic field and electric field, whereas E and H represent the electric and magnetic field intensities, respectively, with time-varying nature.

**Figure 11:**
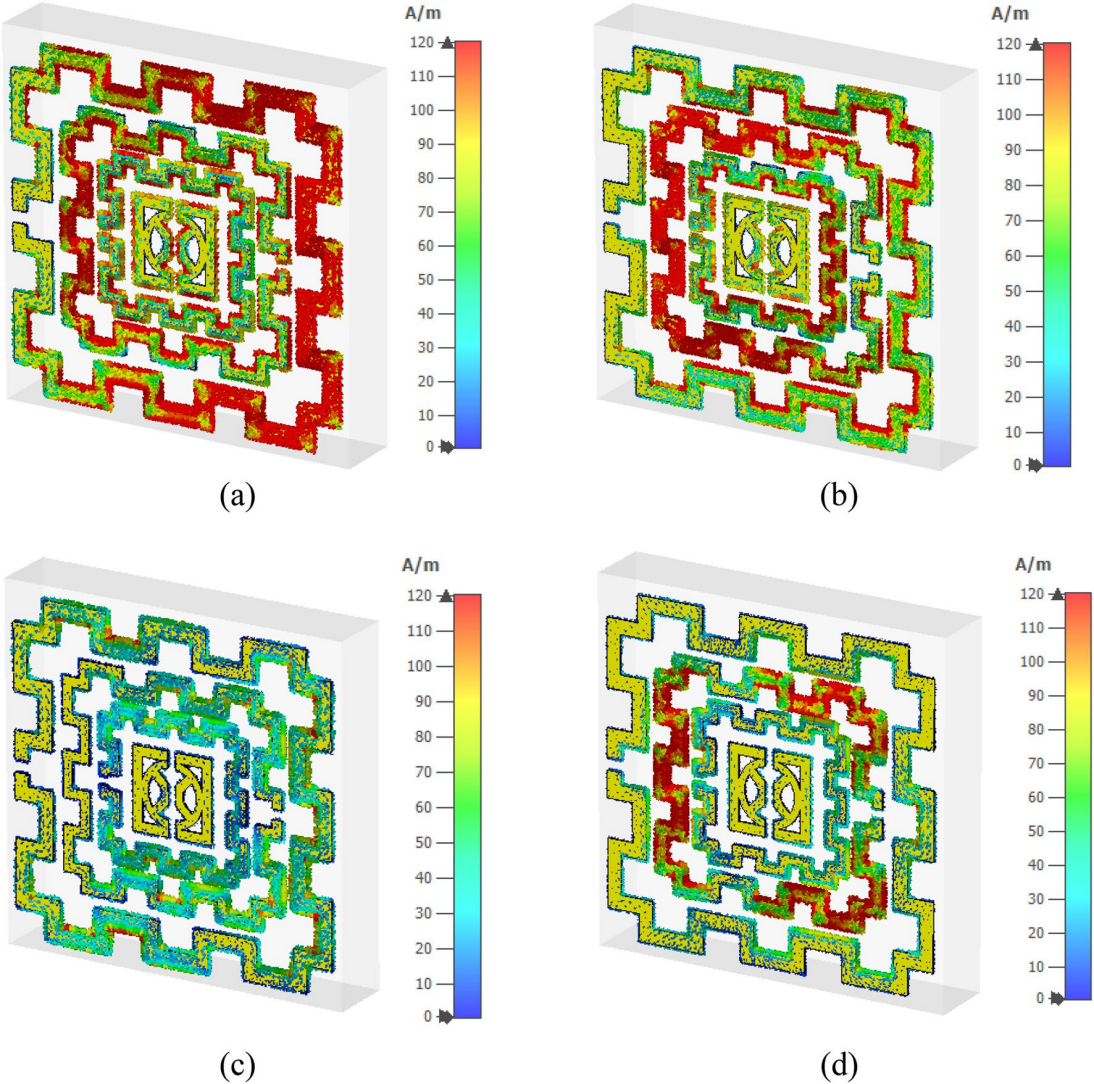
Surface current distribution (**a**) 2.48 GHz (**b**) 4.28 GHz (**c**) 9.36 GHz (**d**) 13.70 GHz. (CST STUDIO SUITE 2019, https://www.3ds.com/products-services/simulia/products/cst-studio-suite)^28^.

The metamaterial's surface current is presented in Fig. 11a–d, in which Fig. 11a represents the same at 2.48 GHz. This Figure reveals that at 2.48 GHz an anticlockwise circular current flows through all rings. At the right half of the first ring and the left half of the second ring, the current density is relatively high, whereas the lower dense current is detected in all other rings. In other inner rings, the current distribution is uneven; a high current is observed at the edge compared to the other portion of the ring. At the resonance of the 4.28 GHz (Fig. 11b), the distribution of electric current is drastically shifted in the outer ring as a moderate amount of current flows through it. A dense current is noticed at various edges of this ring. At this frequency, the current concentration is high in the third ring compared to that of 2.38 GHz. the innermost ring has a lower current concentration. A drastic decreasing current is observed at 9.36 GHz, as shown in Fig. 11c. In that frequency, all the rings contain an average flow of low intense current except the innermost ring, where current is nearly nullified. Meanwhile, as shown in Fig. 11d, surface current intensity again increases at the frequency 13.7 GHz, and the second ring contributes to the maximum amount of the current. In contrast, the third ring exhibits a moderate flow of the current.

**Figure 12:**
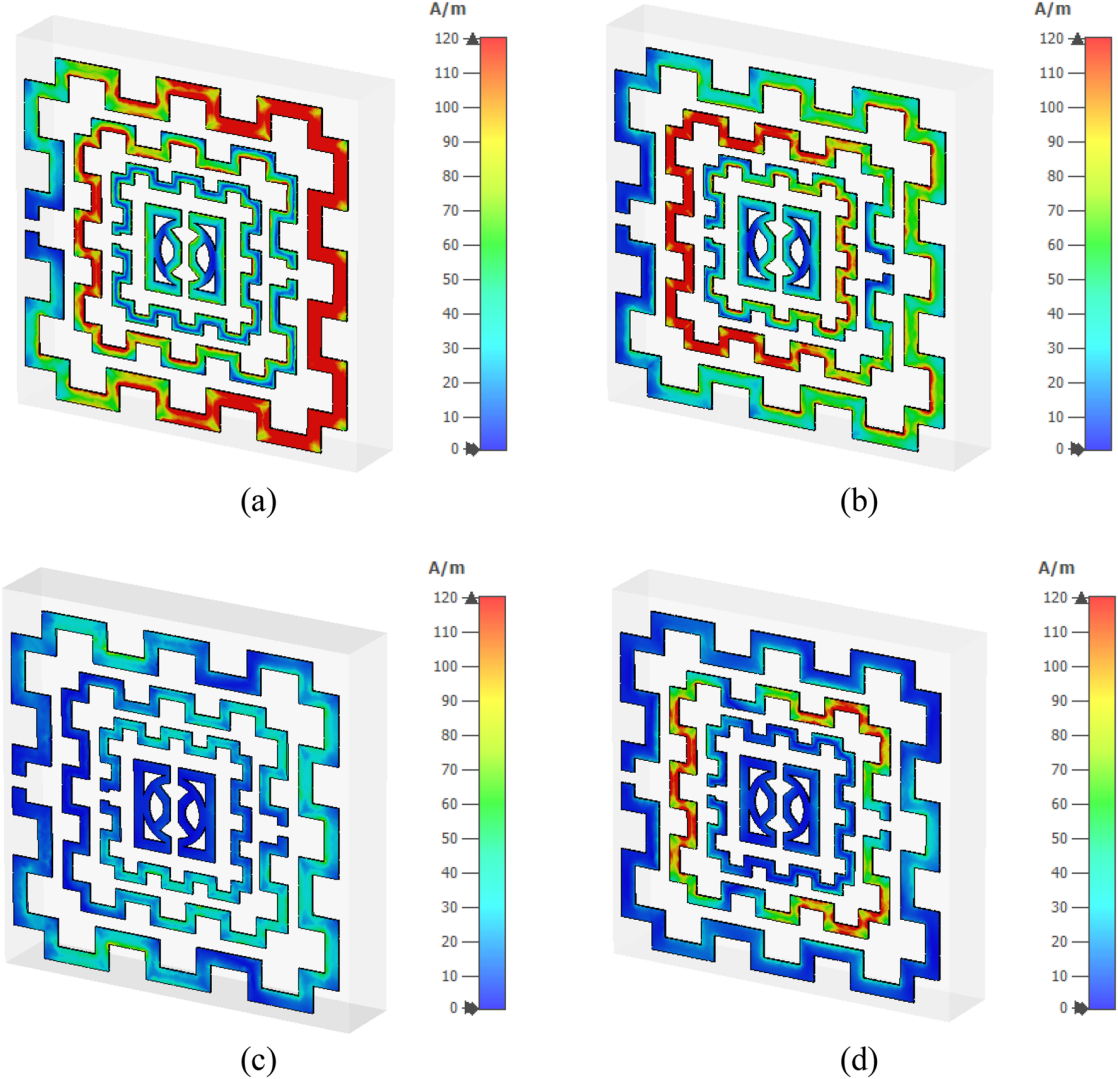
Magnetic field distribution (**a**) 2.48 GHz (**b**) 4.28 GHz (**c**) 9.36 GHz (**d**) 13.70 GHz. (CST STUDIO SUITE 2019, https://www.3ds.com/products-services/simulia/products/cst-studio-suite)^28^.

**Figure 13:**
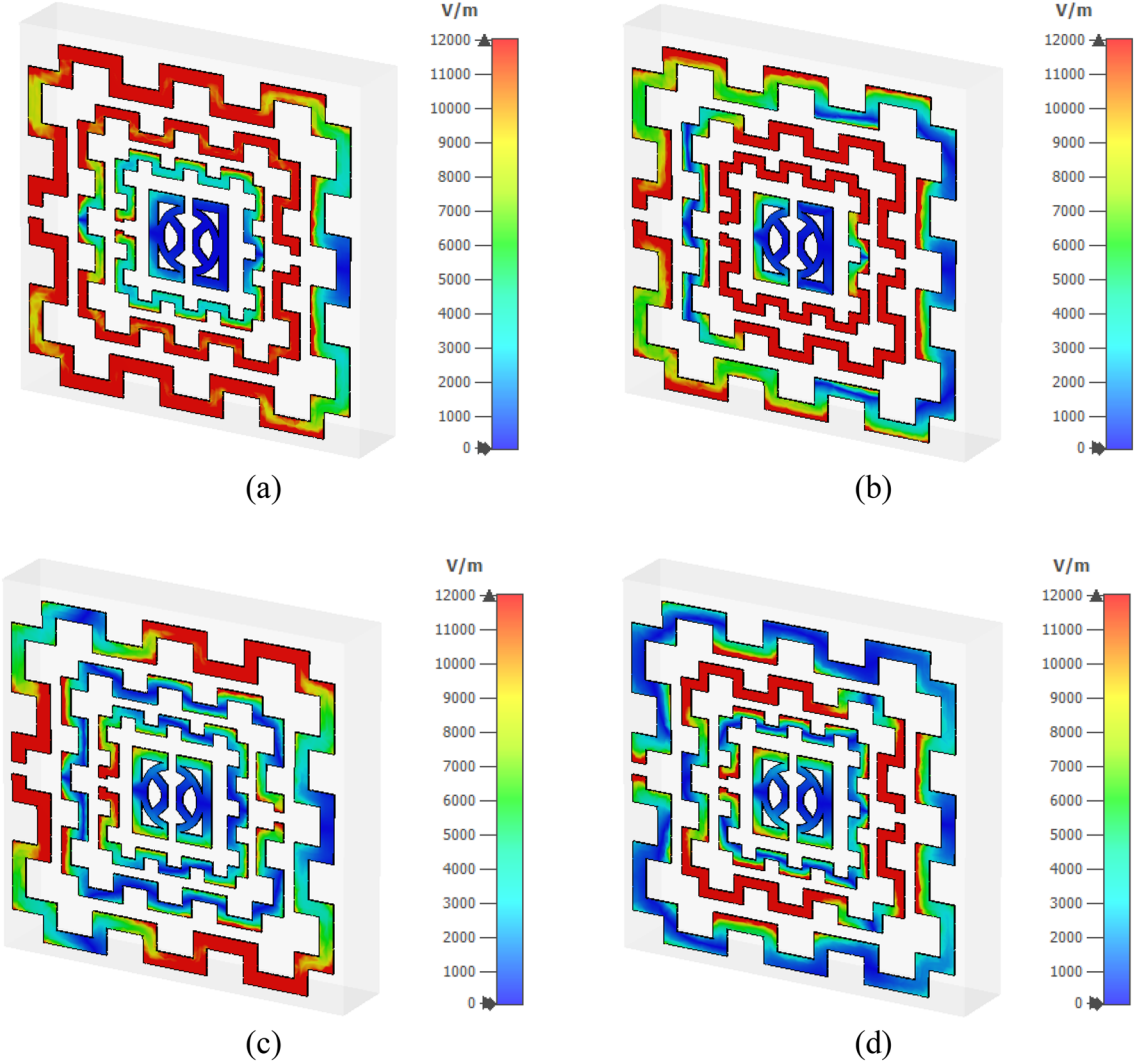
Electrical field distribution at four resonance frequencies (**a**) 2.48 GHz (**b**) 4.28 GHz (**c**) 9.36 GHz (**d**) 13.70 GHz. (CST STUDIO SUITE 2019, https://www.3ds.com/products-services/simulia/products/cst-studio-suite)^28^.

Figure 12a–d shows the magnetic field distribution for four major resonance frequencies. As presented in Eq. (14) magnetic field is interrelated to the surface current density. In Fig. 12a, a high magnetic field intensity is observed at those places where a higher density of current has existed; thus, it satisfies Maxwell’s equation as expressed in Eq. (14). Similarly, a high magnetic field is noticeable at the left half of the second ring as shown in Fig. 12b, whereas all other rings exhibit the magnetic field strength as per the current density. In Fig. 12c, a low-intensity magnetic field is noticed as the current is low at this frequency, whereas in Fig. 12d second ring exhibits significant magnetic field intensity though the field intensity is low through other parts of all other rings. The electric field distribution is presented in Fig. 13a–d. Equation (13) expresses that varying magnetic field has an impact on the induced electric field. A comparison of Fig. 13a with Fig. 12a revealed that an intense electric field is observed at the points where the rate of change in the magnetic field is high. A high electric field is noticed in the vicinity of the gap in the ring due to the capacitive effect. Thus, the outer and second inner ring shows a higher electric field than other rings at 2.48 GHz. Contrarily, in Fig. 13b, at 4.28 GHz, the third ring shows a high electric field since magnetic field variation is more prominent at the same frequency in that ring. At 9.36 GHz, an intense field is noticeable at the split and those portions in the outer ring where the rate of magnetic field variation is high (shown in Fig. 13c). Similarly, as presented in Fig. 13d, the second ring contributes to a significant electric field as magnetic field variation in the same ring is larger (shown in Fig. 12d). Moreover, the electric field is nullified at those parts of the resonator where the magnetic field is constant. Thus, current and electromagnetic fields are closely related to each other, and combinedly contribute to the resonances at the described frequencies.

## Equivalent circuit modeling and analysis

**Figure 14:**
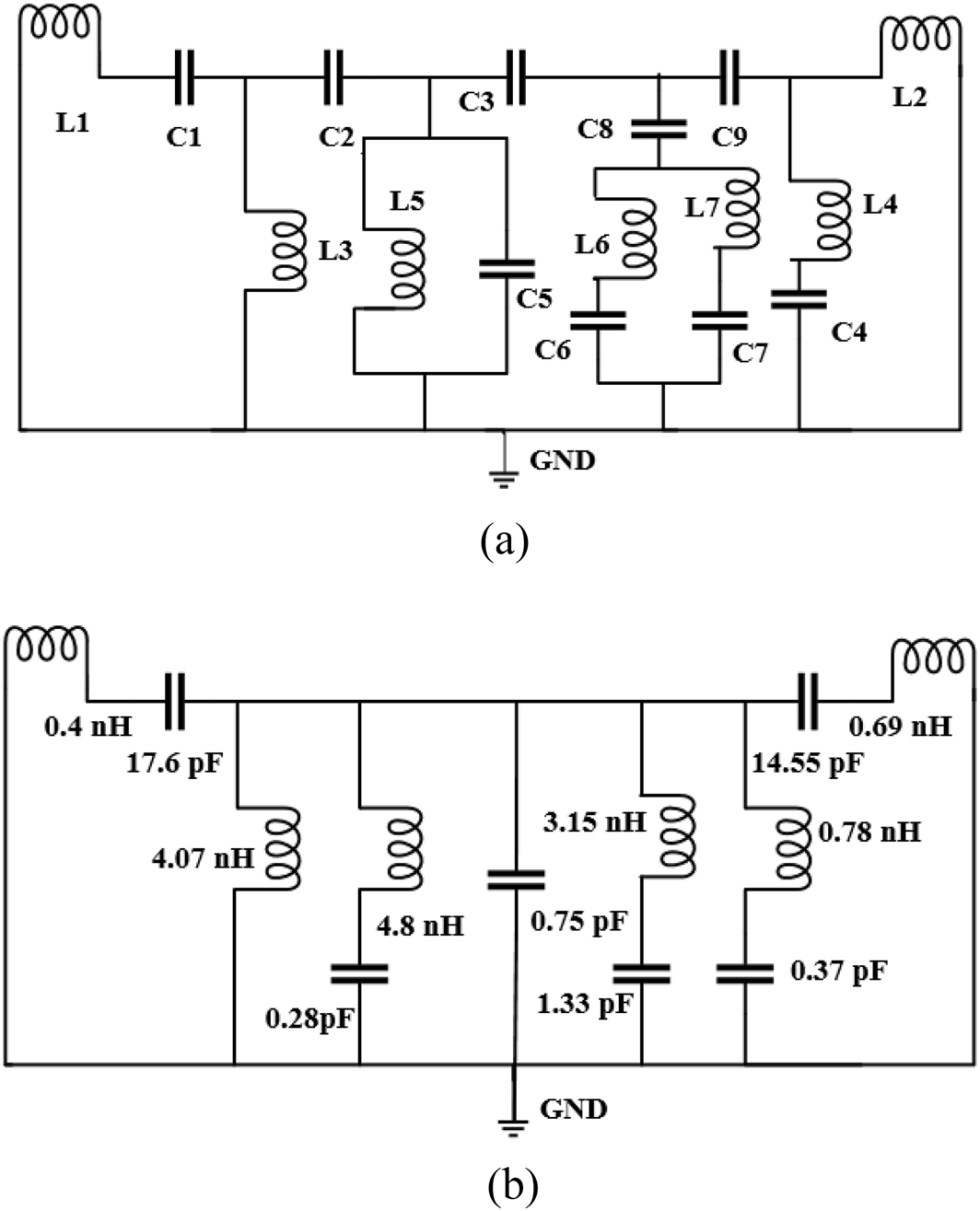
(**a**) Primary equivalent circuit of the proposed unit cell. (**b**) Modified equivalent circuit of the proposed unit cell.

**Figure 15:**
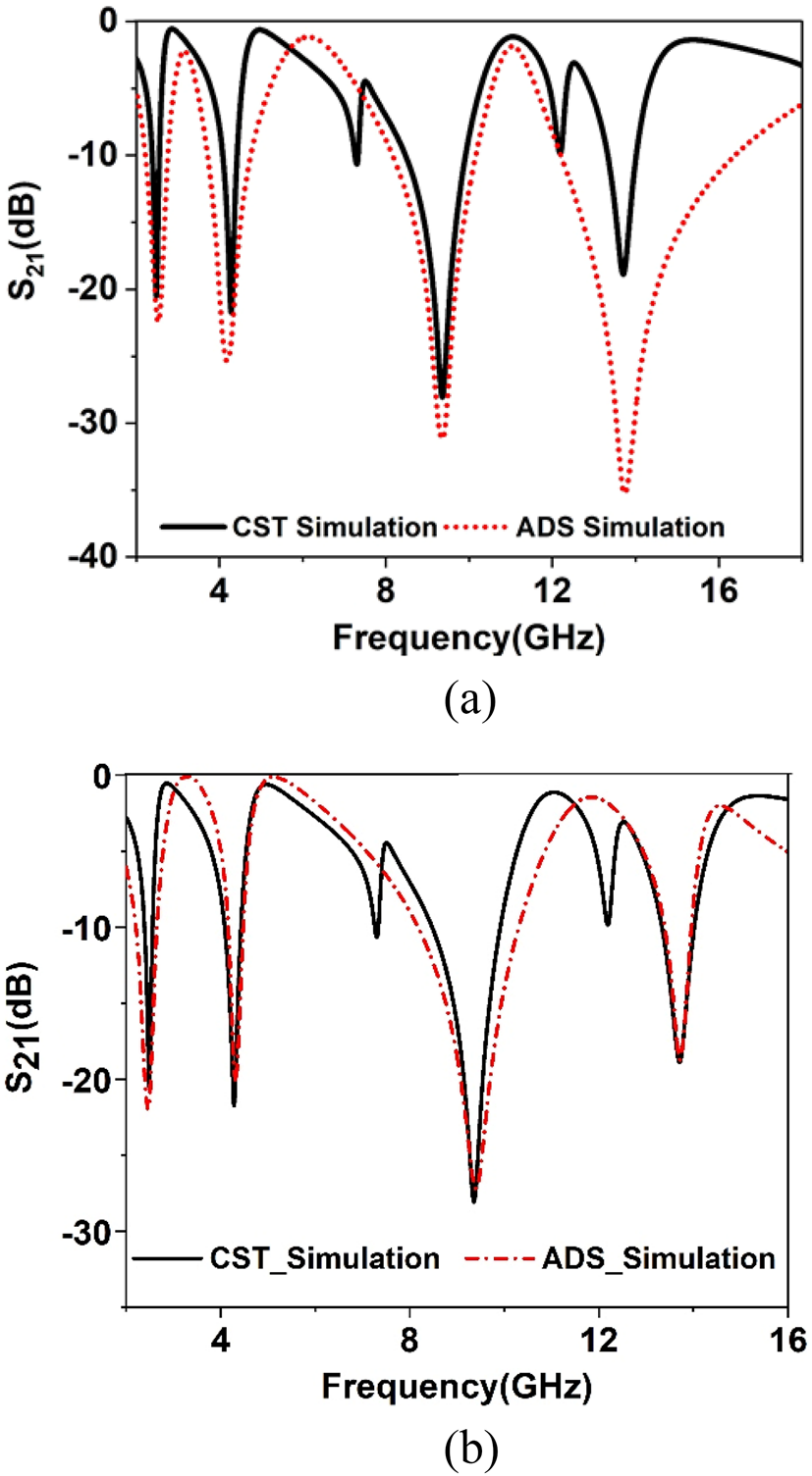
(**a**) Comparison of S_21_ where ADS output obtained for primary equivalent circuit of Fig. 14a. (**b**) Comparison of S_21_ where ADS output obtained for modified equivalent circuit of Fig. 14b.

The researchers have followed numerous approaches to model the equivalent circuit. In^36^, the cavity model approached has been presented where the resonating element can be considered as an RLC tank circuit, whereas the lumped equivalent circuit approach contemplates the microwave elements consisting of inductance, resistance, capacitance, and conductance^37^. The equivalent circuit of the proposed metamaterial unit cell can be designed by considering the metallic conductor with inductor property since, owing to the current flow, magnetic induction occurs. The split present in each of the ring exhibits capacitive effect; thus, every spit ring resonator acts as the resonant tank circuit with inductance L and capacitance C. thus, the split ring acts as a resonator showing resonance at a specified frequency, and it can be controlled by precise control of L and C values with the help of controlling length and thickness of the ring and also controlling the split gap and inter-ring distance. The primary equivalent circuit of the proposed unit cell is shown in Fig. 14a. In this equivalent circuit, Inductances *L1*, *L2* in the corporation of capacitance, *C1* represent equivalent circuit elements of the outermost split ring.Meanwhile, inductor pair *L3* and *L4* represent the equivalent inductance of the second ring, whereas element *C4* is its capacitance formed by the split gap. On the other hand, inductor-capacitor pair *L5* and *C5* is the contribution of the third ring to the equivalent circuit, whereas inductances and capacitances of the innermost ring are displayed by the components *L6, L7, C6,* and *C7*. The coupling between one ring to another is represented by the coupling capacitors *C2, C3, C8*, and *C9*. The values of these circuit components are obtained by using simulation software Advanced design system (ADS)-2016^38^ considering S_21_ is the target response and listed in Table 2. By tuning the component values in ADS, the values are so chosen that it provides similar resonances of S_21_ obtained from the CST. Figure 15 shows the S_21_ plot that displays this quantity for both CST and ADS simulation. The results show a close similarity with each other though ADS output deviates from the CST in the sense of two small resonances near 7.5 and 12 GHz. This discrepancy is because while drawing the ADS circuit, the effect of mutual inductance and parasitic capacitance is neglected for the simplicity of the equivalent circuit. A more significant deviation in magnitude is obtained at the resonance of 13.7 GHz, which can be adjusted by considering the resistive effect of the inductance *L7*, that triggers the resonance at this frequency. The effects of these circuit components are studied carefully. The study reveals that L1 and C1 predominantly influence the resonance frequency of 4.28 GHz, the resonance frequency of this band can be adjusted by controlling the values of these elements.Meanwhile, it is observed that inductance and capacitance pair L7 and C7 show their influence over the resonance of 2.48 GHz, which can be tuned by changing the value of these elements. Parallelly connected components L5 and C5 also exhibit their influences over the resonance in 2.48 GHz, those can also be used to modify the shape of the S_21_ waveform. The amplitudes of resonance at 2.48 GHz and 4.28 GHz can also be modified by changing the inductances L2 and L3. The emergence of the resonance at 13.7 GHz is supported by inductor L4 and capacitor C4, whereas the branch having the components L6 and C6 is responsible for the resonance at 9.36 GHz. The remaining components can be tuned to adjust the magnitude of total S_21_. The equivalent circuit can be further modified to obtain a more simple circuit that provides more proper matching between the result obtain form ADS and CST. This modified equivalent circuit is depicted in Fig. 14b in which mutual coupling terms are neglected and the mtm equivalency is expressed with a number of parallel branches having inductors and capacitors. The deviation between ADS and CST simulation of Fig. 15a is eliminated by proper tuning of the component values of the modified circuit ( shown in Fig. 14b). The S_21_ obtained from this modified circuit is compared with the CST result that shows the perfect matching of the resonances for the desired four frequency of resonances as depicted in Fig. 15b.

**Table 2:** Circuit components and values of the primary equivalent circuit.

Capacitor	Value (pF)	Capacitor	Value (pF)	Inductor	Value (nH)	Inductor	Value (nH)
C1	0.1	C2	4.97	L1	1.38	L2	4.59
C3	7.72	C4	0.5	L3	9.05	L4	0.27
C5	0.05	C6	0.37	L5	6.49	L6	0.79
C7	1.0	C8	8.1	L7	4.02	–	–
C9	2.78	–	–	–	–	–	–

## Result and discussion

In this section, the metamaterial property of the proposed MTM is extracted, and analysis has been done on the obtained result to identify the characteristic of the intended design. The measurement result is also included in this section, along with the comparison and discussion. Since most of the time, metamaterial works in the array, for this reason, the performance of different arrays of this metamaterial is also required to analyze. The frequency hopping characteristics has been studied through numerical simulation by using switches at splits gaps of the outer three rings. The performance of the metamaterial is further compared with some recently published work in this section.

### Unit cell property extraction and analysis

**Figure 16:**
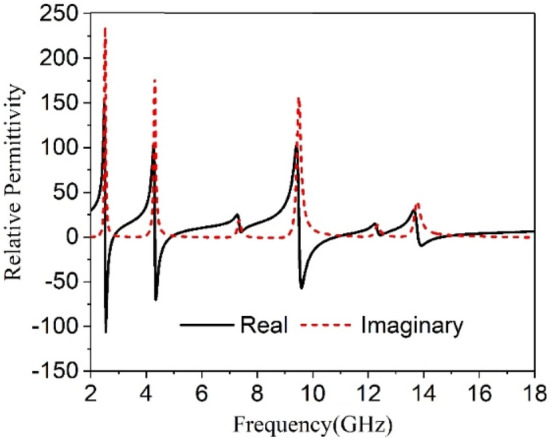
Real and Imaginary part of relative permittivity.

**Figure 17:**
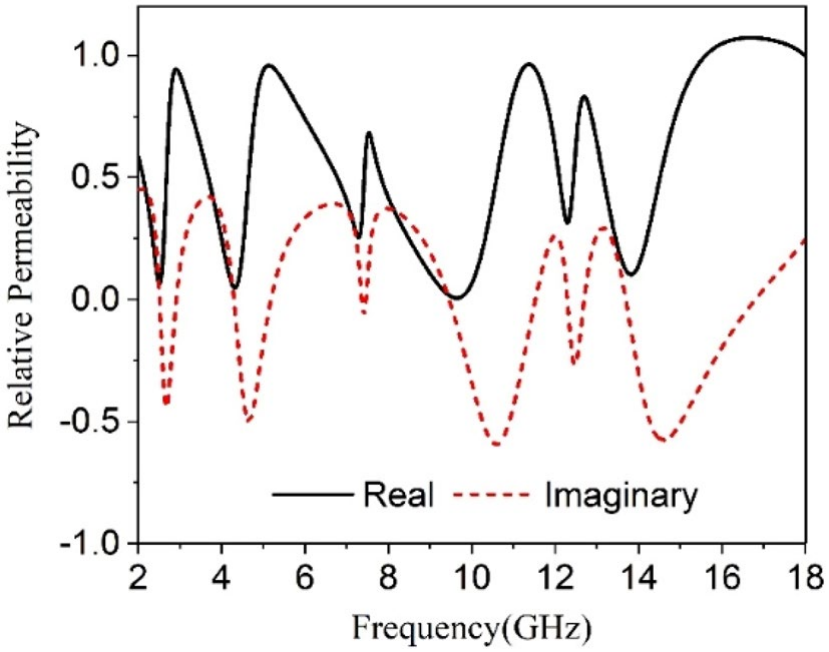
Real and Imaginary part of relative permeability.

**Figure 18:**
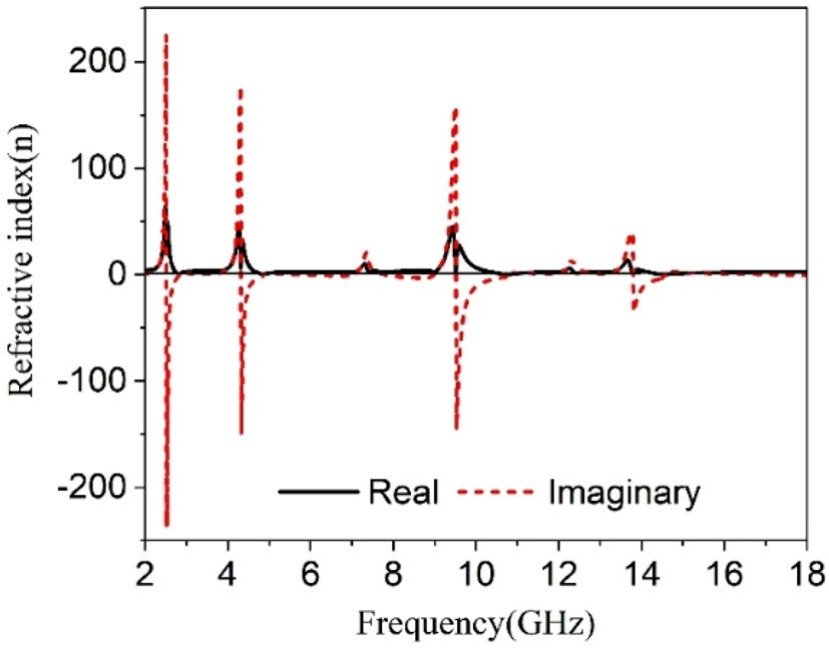
Refractive index of proposed unit cell.

**Figure 19:**
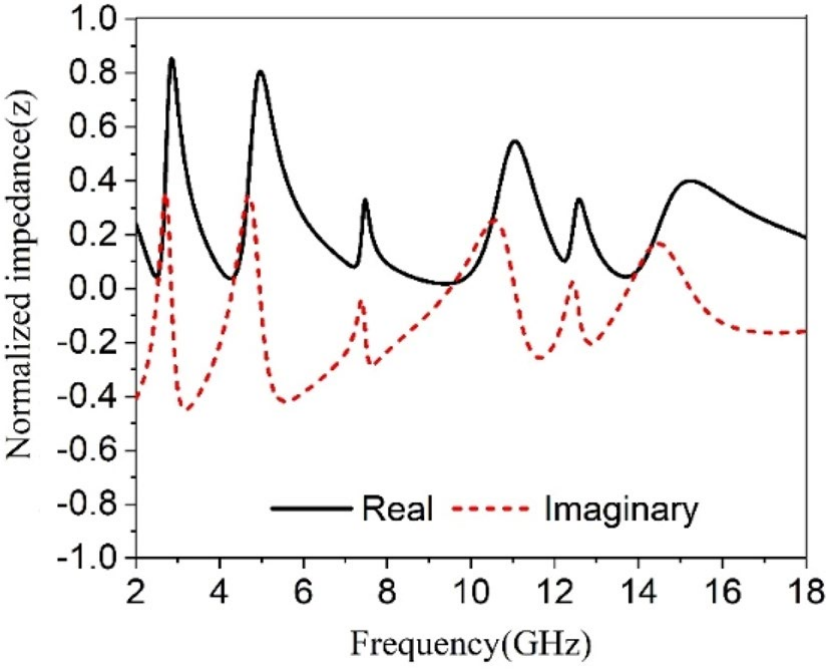
Normalized impedance of the proposed unit cell.

The transmission coefficients (S_21_) and reflection coefficient (S_11_) are attained from the CST microwave studio has been already presented in Figs. 5 and  6. These results have been utilized in MATLAB code based on Eqs. (8)–(10) to get relative permittivity, permeability, refractive index, and impedance. The results obtained from this code are used to plot the graphs of these parameters and displayed in Figs. 16, 17, 18 and 19. Figure 16 shows the permittivity graph that shows negative permittivity in four distinct ranges of frequencies of 2.51–2.81 GHz, 4.3–4.9 GHz, 9.51–10.9 GHz, and 13.8–14.6 GHz, respectively. Two other resonances are also observed near 7.43 GHz and 12.44 GHz, but these resonances do not contribute to negative permittivity. The imaginary parts of the permittivity are positive thus it satisfies the criteria of metamaterial behavior. Contrary to this, the permeability graph plotted in Fig. 17 shows positive permeability characteristics all over the frequency range of consideration where permeability fluctuates within 1–0. Near zero permeability property is noticed from this graph with a minimum value of permeability of 0.06, 0.04, 0.001,0.09 2.52, 4.32, 9.67, 13.84 GHz, respectively. Two other lower peaks are also noticed at 7.3 GHz and 12.3 GHz. Within these later one shows a relatively high value compared to the previous minima. The refractive index plot is presented in Fig. 18 that exhibits a near-zero refractive index with minimum indices of 0.07 at 2.8 GHz, 0.007 at 4.86 GHz, 0.01 at 10.76 GHz, 0.01 at 14.5 GHz. Similarly, the normalized impedance of the proposed MTM unit cell shows less than unity impedance for all over the frequency ranges of interest, with the real part is always positive as expressed in Fig. 19. Near zero impedances are observed near the resonance frequency of S_21_. The outcomes of MTM are also summarized in Table 3. Positive real impedance indicates that the proposed metamaterial acts as a passive medium. It is also a noticeable fact from the refractive index plot of Fig. 18 that the imaginary part of the refractive index is positive in the region of 2–2.52.5–2.81 GHz, 2.81–4.32 GHz, 4.85–5.8 GHz, 7–7.8 GHz, 8.98–9.5 GHz, 10.77–13.8 GHz, and 14.57–18 GHz respectively. The existence of a positive imaginary value of the refractive index makes the incident wave decreasing inside of the structure. Single negative metamaterial with near-zero refractive index exhibits its potentiality to increase the gain and directivity of the antenna^16,39,40^. The proposed MTM is applied with this antenna as a superstrate to observe the gain characteristics of the antenna.

**Table 3:** Extracted data for different properties of the proposed MTM unit cell.

Parameter	Bandwidth (GHz)	Frequency of resonance/minimum value	Criteria (Bandwidth)
S_21_ (magnitude)	2.4–2.59, 4.1–4.5, 8.7–10, 13.4–14	2.48, 4.28, 9.36, 13.7	S21 < −10 dB
S_11_ (magnitude)	2.75–3, 4.75–5.26, 10.74–11.35, 12.5–12.64, 14.8–15.4	2.85, 4.95, 11.1, 12.55, 15	S11 < −10 dB
ε_r_ (real)	2.51–2.81, 4.3–4.9, 9.51–10.9, 13.8–14.6	2.54, 4.34, 9.6, 13.9	ε_r_ (real) < 0
µ_r_ (real)	2.4–2.6, 4–4.5, 8.67–10.28, 13.5–14.17	2.52, 4.32, 9.67, 13.84	µ_r_ (real) < 0.2
n (real)	2.78–2.84, 4.78–4.95, 10.57–10.98, 14.34–14.9	0.07, 0.01, 0.01, 0.01	n < 1
Z (real)	2–2.72, 3.13–4.7, 5.42–10.87, 11.23–18	2.5, 4.25, 9.4, 13.7	Z < 0.5

**Figure 20:**
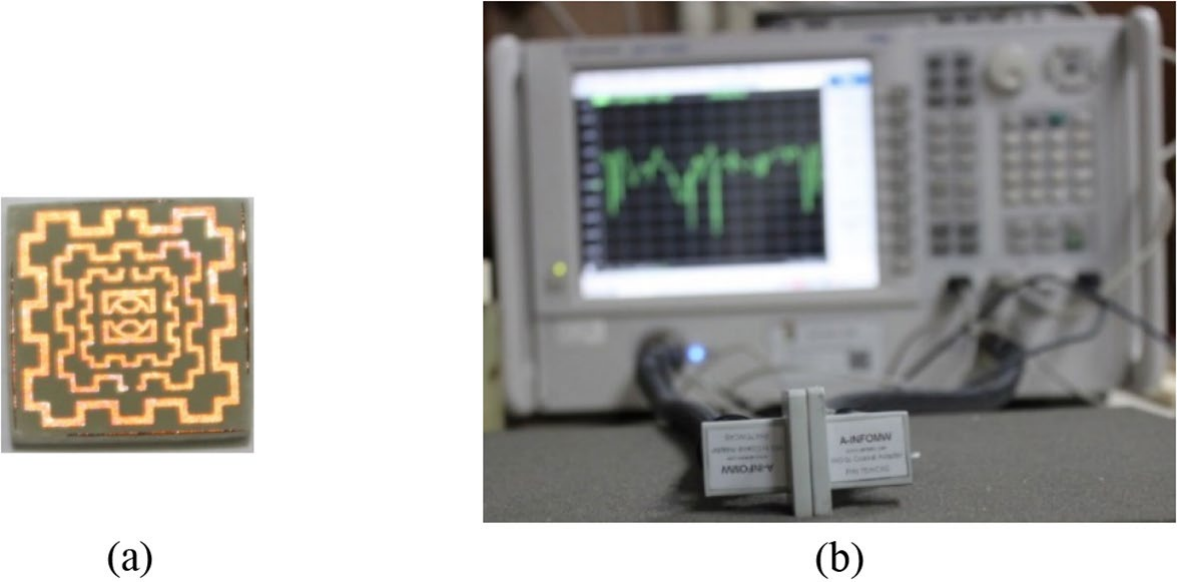
(**a**) Fabricated unit cell (**b**) transmission coefficient measurement setup.

**Figure 21:**
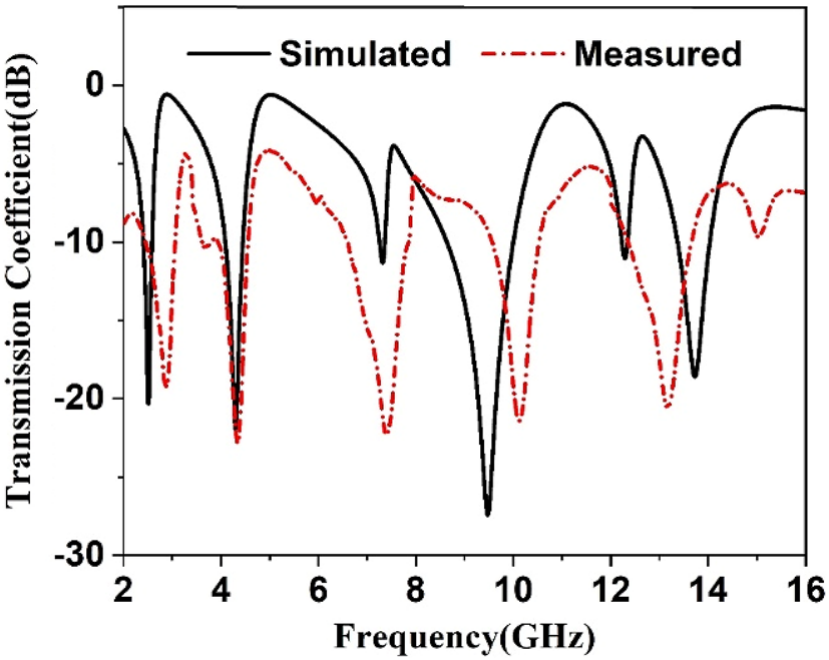
The measured transmission coefficient of the proposed MTM unit cell.

The metamaterial performance is further studied by fabricating the prototype of the proposed metamaterial unit cell. Figure 20a shows the fabricated prototype of the proposed MTM unit cell whereas Fig. 20b exhibits the measurement setup. In the measurement process, a close boundary condition is applied where the prototype is placed in between two waveguide ports. One of the waveguides acts as a transmitter whereas another one acts as a receiver. The waveguide ports are connected to a vector network analyzer (VNA). The measurement is taken from 2 to 16 GHz and the measured result is shown in Fig. 21. From this Figure, it is observed that measured S_21_ is well-matched with the simulation result. A little deviation in measured and simulation results is observed at resonances of 2.4 GHz, 9.5 GHz, and 13.7 GHz. It is also noteworthy to mention that measured results inherit some amount of noise and harmonics along with the magnitude variation compared to the simulation results. Fabrication errors, coupling effect of the two waveguide ports have some impact on this deviation between and simulation results.

### Array metamaterial analysis

**Figure 22:**
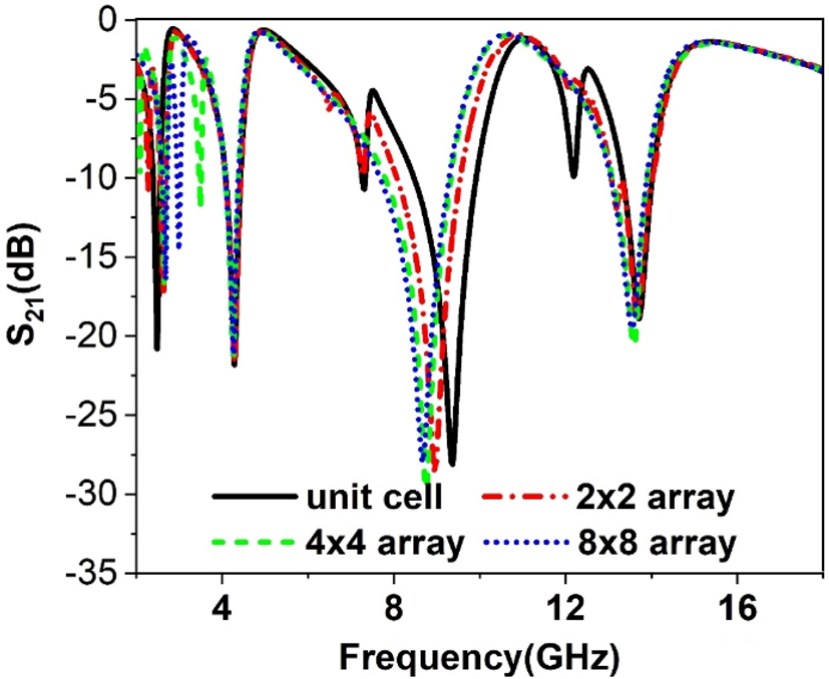
S_21_ comparison between the unit cell and different arrays.

Since in most cases, an array of unit cells is used together instead of a single unit cell, the performance of the array of the unit cells is observed considering 2 × 2, 4 × 4, and 8 × 8 arrays of the unit cells. The array performance is investigated utilizing the simulation setup arranged in the same manner as the unit cell simulation. Figure 22 shows the S_21_ plot for these three arrays. A comparison with the unit cell reveals that the resonance 2 × 2 array exhibits two resonances around 2.5 GHz with a shift in frequency and amplitude. The second and last resonances are well-matched in this case, whereas the third resonance has a mismatch with shifting the resonance frequency towards the lower frequency. Meanwhile, in the case of a 4 × 4 array, within 2–4 GHz, three resonances are observed though resonance frequency within 2–6 GHz is unaltered. The resonance frequency occurred with 8–10 GHz shifts its position towards lower frequency compared to the response of 2 × 2 array. Moreover, A slight shift in resonance frequency and amplitude is also noticeable in resonance within 12–14 GHz. Lastly, the performance of the 8 × 8 array is investigated, whose resonance frequency is nearly coexisted with the 4 × 4 array with dissimilarity in low-frequency resonance. It is evident from this study that all the arrays exhibit a minor mismatch with the unit cell output though they all cover S, C, X, and Ku bands. The origin of mismatching in outcomes among different arrays and the unit cell can be investigated by analyzing the electric field, magnetic field, and surface currents.

**Figure 23:**
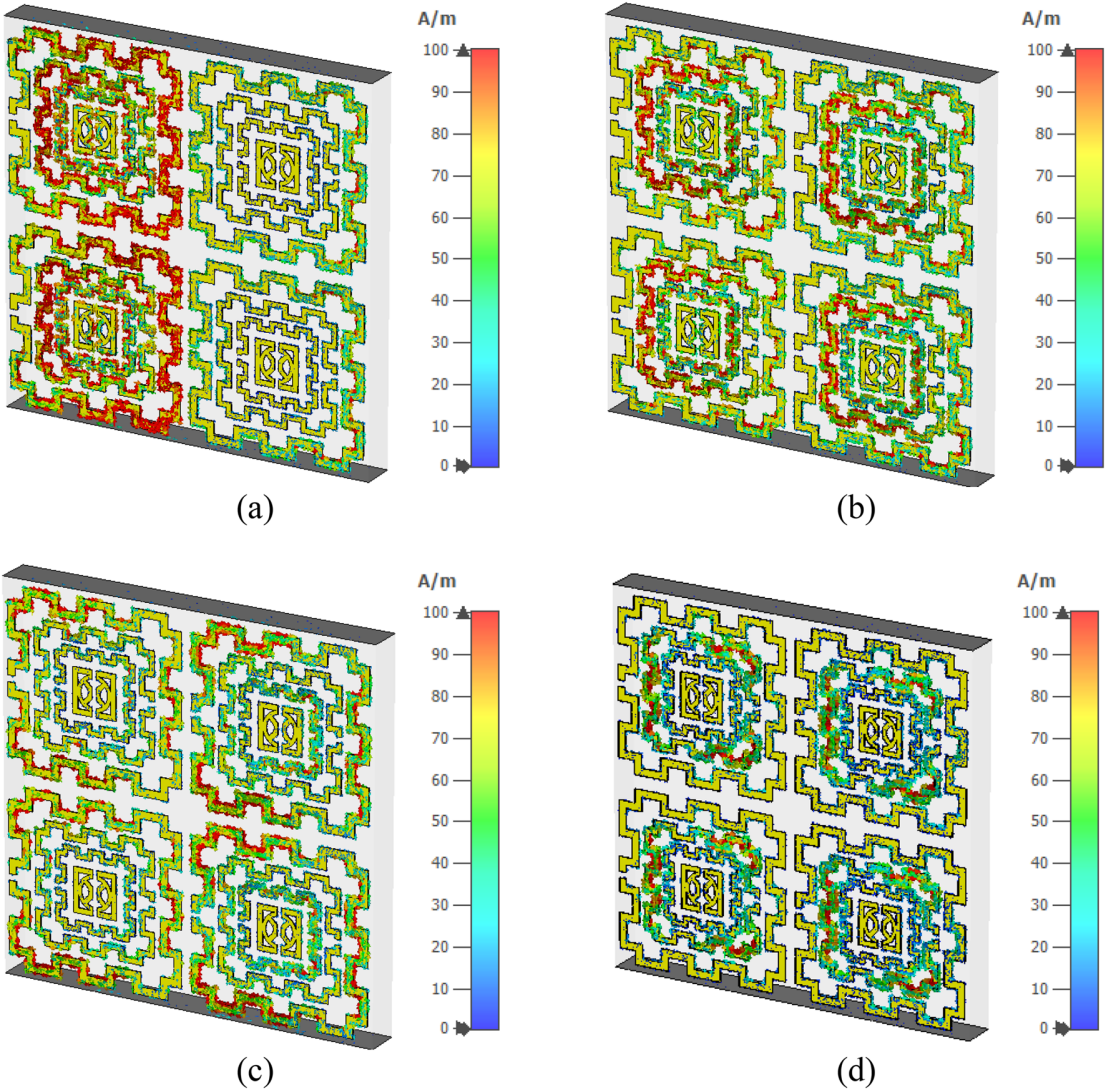
Surface current analysis of 2 × 2 array at (**a**) 2.63 GHz, (**b**) 4.28 GHz (**c**) 8.9 GHz, and 13.7 GHz. (CST STUDIO SUITE 2019, https://www.3ds.com/products-services/simulia/products/cst-studio-suite)^28^.

**Figure 24:**
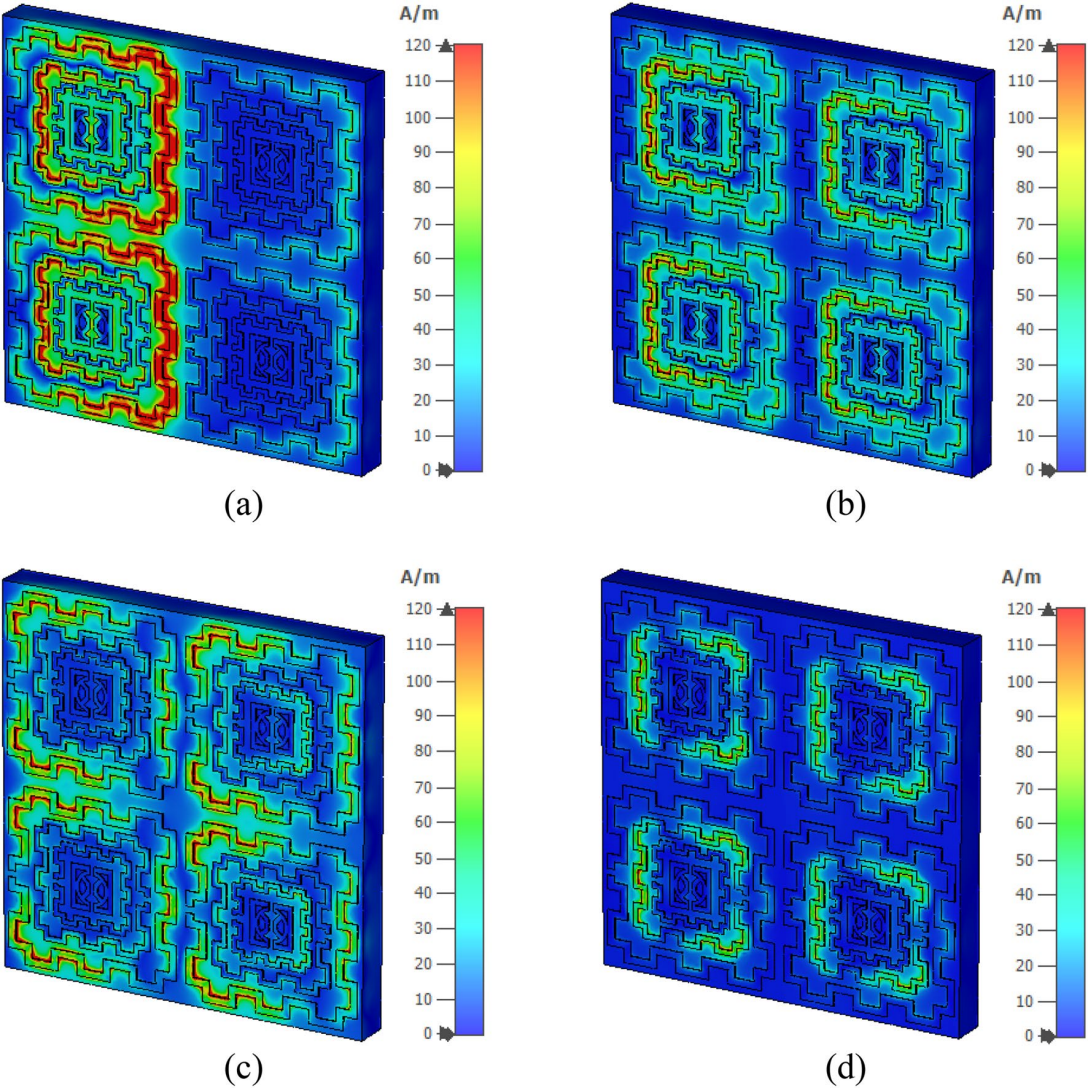
Magnetic field distribution of 2 × 2 array at 2.63 GHz, (**b**) 4.28 GHz (**c**) 8.9 GHz, and 13.7 GHz. (CST STUDIO SUITE 2019, https://www.3ds.com/products-services/simulia/products/cst-studio-suite)^28^.

**Figure 25:**
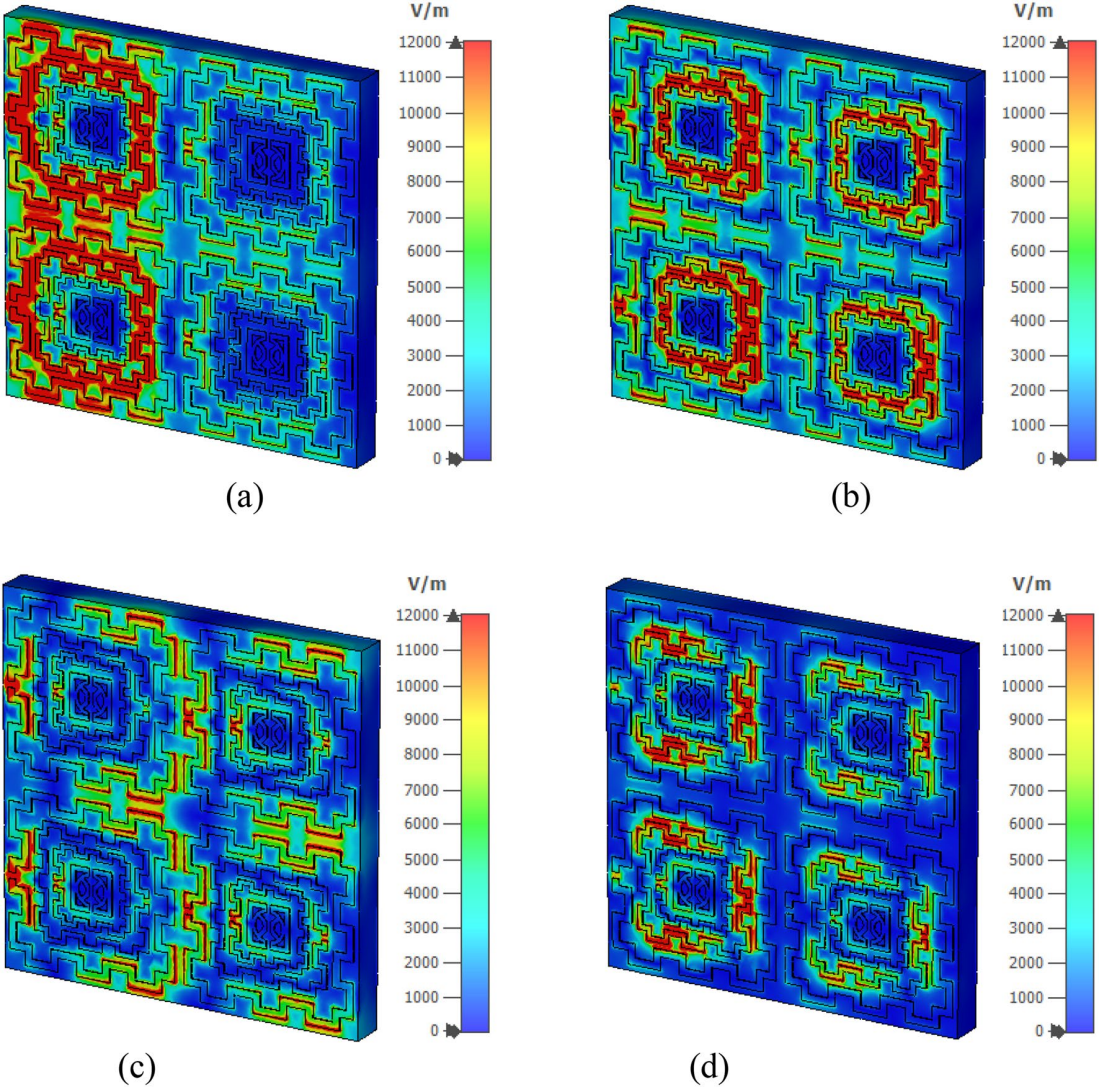
Electric field distribution in 2 × 2 array at (**a**) 2.63 GHz, (**b**) 4.28 GHz, (**c**) 8.9 GHz, and (**d**) 13.7 GHz. (CST STUDIO SUITE 2019, https://www.3ds.com/products-services/simulia/products/cst-studio-suite)^28^.

Figure 23 shows the surface current distribution of the 2 × 2 array. From this Figure, it is observed that unit cells of the left column in the array contain a large concentration of current in their outer ring as shown in Fig. 23a. This large current is inducing a magnetic field that creates a strong coupling between two array elements, as shown in Fig. 24a. A high electric field is also noticed between two vertical unit cells of the first column in which the electric field of two adjacent unit cells overlapped to each other, as exhibited in Fig. 25a. So, the mutual coupling is obvious around 2.5 GHz, which causes two resonances, one at 2.29 GHz and another at 2.63 GHz. Both resonances are shifted from the unit cell resonance of 2.48 GHz. At, 4.28 GHz a moderate amount of current flows through all unit cells, as shown in Fig. 23b. Meanwhile, a small induced magnetic field is observed in Fig. 24b with no mutual coupling effect among the unit cells. Though Fig. 25b shows a small mutually coupled electric field between two cells of the first column, it has a negligible effect on the array's performance.For this reason, no shift in resonance is noticed in terms of frequency. The magnetic field distribution showed in Fig. 24c reveals that at 8.9 GHz, the mutually induced magnetic field exists among the two adjacent cells of the first column. It causes a moderate amount of current flow through the corresponding unit cells' outer rings (shown in Fig. 23c). In Fig. 25c, the adjacent cells' electric field is coupled to each other. Thus, electric and magnetic field coupling occurs simultaneously that causes to shift of resonance frequency towards a lower value compared to the unit cell resonance. Lastly, surface current, magnetic, and electric field distribution at 13.7 GHz is presented in Figs. 23d, 24d, and 25d, respectively. No mutual coupling in an electric and magnetic field is observed in this frequency.

Moreover, the current through the outer ring of the unit cells is not significant. Thus, no noticeable change in the resonance is observed at this frequency. From this study, it can be concluded that in the 4 × 4 and 8 × 8 array, this coupling effect will be more pronounced and complex as more unit cells are involved, which causes to shift in the resonance frequencies. Thus, the transmission coefficients of 4 × 4 and 8 × 8 arrays suffer from a deviation in resonance frequency and magnitude, as described in Fig. 22.

### A numerical study on frequency hopping through switching

**Figure 26:**
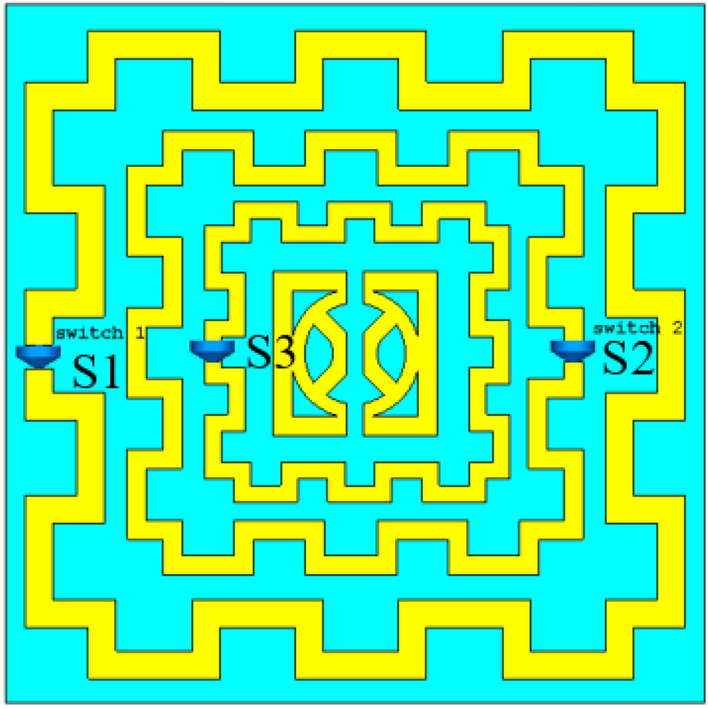
Open and short circuit of the split gaps through the switches S1, S2, and S3. (CST STUDIO SUITE 2019, https://www.3ds.com/products-services/simulia/products/cst-studio-suite)^28^.

**Figure 27:**
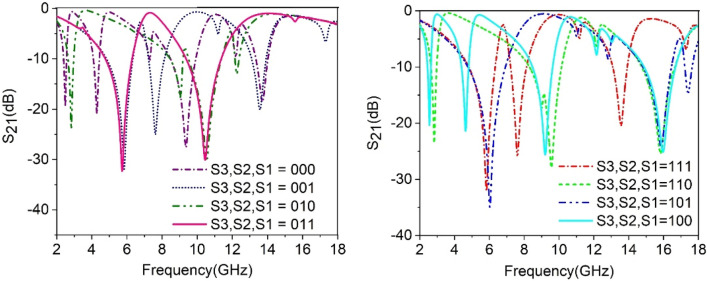
Frequency hopping by using switches at different split gaps of the resonators.

The effect of split gap distance on the performance of the metamaterial has been already studied in Sect. 3. In this section, a numerical study is accomplished here to obtain multiband resonances in which the band of resonances can be shifted from one frequency region to another through the open and short-circuited of the split gaps of three outer rings using three switches S1, S2, and S3 as shown in Fig. 26. The effect of this switching is observed in S_21_ performance of the proposed MTM that provides eight different sets of the frequency bands for 2^3^ = 8 different states of the open and short circuit in the split gaps of the outer three rings. By sequential changing of the states of the switches from ON to OFF transforms the resonance phenomena from one frequency domain to another because when the split gap is shorted by closing the switch with near-zero resistance, the capacitive effect due to that split gap is eliminated, and the ring becomes a closed ring which has only inductive effect. Thus the resonance frequencies of the MTM are altered by changing the status of the switches. Figure 27 shows the transmission coefficient results for different switching configurations in which binary 0 represents the open state, whereas binary 1 resembles the short circuits at the split gaps. The resonance frequencies and covering of different bands for different states of open/short-circuited of the split gaps are also presented in Table 4. In the case of the proposed MTM, where the outer three ring’s split gaps are remained undisturbed due to switching with a switching state of S1, S2, S3 = 000, S_21_ covers S, C, X and Ku bands with four resonances at 2.48, 4.28, 9.36, 13.7 GHz. If switch S1 is closed, that makes a short circuit in the split gap of the outer ring to eliminate the capacitive effect of the outer ring. The effect of shorting the gap of the outer ring changes the total resonance phenomena due to the change of the total capacitance and inductance of the MTM unit cell, and three major resonances are observed at 5.85, 7.6, and 13.6 GHz, respectively, covering C and Ku bands. In the next step, when switch S2 of the second outmost ring is closed, keeping unaffected by the other split gaps, four major resonances are observed near 2.9, 7, 10.8, 12.5 GHz covering S, C, X, and Ku bands. This switching effect is noticed with the transformation of the capacitive effect of second ring to the inductive one that causes a change in resonance frequencies compared to the result obtained by the proposed MTM without any switch. A study of the effect of closing the switch S3 only that makes a short circuit in the split gap of third ring providing four resonances occurred at 2.6, 4.6, 9.2, 15.9 GHz. The effect is noticed with a significant shift in frequency of resonance at high frequency whether the low and midfrequency resonances are less affected. This result indicates that high-frequency resonance is greatly influenced by the split gap of the third ring. Now, the effect of the shorting of the two split gaps simultaneously is examined through numerical simulation. When the split gaps of the first two outer rings are shorted through the closing of switch S1 and S2 simultaneously, the resonance phenomena due to the outer two rings are affected again due to the elimination of the capacitive elements, and these two rings act as a conductive medium. Due to the outer two rings' inductive effects, two sharp resonances are noticed at 5.7, 10.75 GHz, covering C and X bands. On the other hand, closing the switches S2 and S3 cause the elimination of the capacitances exerted by the second and third rings. With the more pronounced inductive effects of these two inner rings along with the impact of the other rings, the metamaterial exhibits three resonances at 2.8, 9.5, 15.8 GHz covering S, X, and Ku bands. Similarly, the effect of closing the switches S1 and S3 causes three resonances at 6, 15.8, 17.2 GHz. When all switches are closed, all three rings act as the inductive elements; in that case, intra-ring capacitance plays a vital role in forming the LC resonance circuit that causes three resonances at 5.8, 7.7, and 13.8 GHz. Thus, open and short-circuited of the three outer rings split gaps by using three switches helps to modify the resonance frequencies offered by the proposed MTM with the flexibility to switch one set of resonance frequencies to another by closing appropriate on–off switches. Thus, desired frequencies can be selected by appropriate open and short circuits of the split gaps, increasing the potentiality of the proposed MTM for various multiband microwave applications.

**Table 4:** Resonances and covering bands of the proposed MTM unit cells for switching positions. S1, S2, and S3 indicate three open, and short circuit switches across the three outer rings split gaps.

S3	S2	S1	Resonance Frequencies	Covering Bands	S3	S2	S1	Resonance Frequencies	Covering Bands
0	0	0	2.48, 4.28, 9.36, 13.7	S,C, X, Ku	1	1	1	5.8, 7.7, 13.8	C and Ku
0	0	1	5.85, 7.6, 13.6	C, Ku	1	1	0	2.8, 9.5, 15.8	S, X, Ku
0	1	0	2.9, 7,10.8, 12.5	S,C, X, Ku	1	0	1	6, 15.8, 17.2	C, Ku
0	1	1	5.7, 10.75	C and X	1	0	0	2.6, 4.6, 9.2, 15.9	S,C,X, Ku

### Performance comparison of the MTM

The performance of the MTM is now compared with some existing works considering the physical dimension, compactness of the unit cell, the number of resonances and covering bands, application areas. The comparison is presented in Table 5. The metamaterial compactness is evaluated by measuring EMR, which can be determined by dividing the wavelength at lower resonance frequency by the unit cell's Maximum physical dimension. The proposed metamaterial exhibits a high EMR of 15.1, making it more compact compared to the other works presented in Table 5. It is observed from the table that, Ref.^41,42^ has the lowest dimension compared to our proposed MTM, but they cover only two bands. Moreover, EMR values presented by them are also low compared to our proposed design. Moreover, in^42^, no particular application is mentioned, whereas^41^ offers multiband applications. In Ref^17,18,43^, metamaterials are exercised to enhance antenna properties, but these materials are comparatively larger in dimension with low EMR. The proposed metamaterial possesses a small size, Quad-band resonances with high EMR making the proposed design superior compared to all other works presented in the comparison table. Due to its compactness and multiband property this metamaterial can be used for various applications of small sized devices.

**Table 5:** Comparison of proposed MTM with recent works in terms of dimension, resonances, covering bands, EMR, and applications.

References	Year	Physical and Electrical dimension	Resonance frequencies (GHz)	Covering bands	EMR	Applications
^15^	2020	9 × 9 0.124λ × 0.124λ	14.93, 10.84, 4.15	C, X, Ku	8.03	Satellite, Radar communications
^17^	2019	15.6 × 15.6 0.14λ × 0.14λ	2.65, 4	S, C	7.3	Antenna mutual coupling suppression
^18^	2020	8 × 8 0.070λ × 0.070λ	3.5	S	10.7	Antenna decoupling
^41^	2017	6 × 6, 0.09λ × 0.09λ	4.3, 7.6, 9.8	C, X	11.5	Multi band application
^42^	2017	5 × 5, 0.23λ × 0.23λ	13.9,27.5	Ku, K	4.4	Not mentioned
^43^	2020	8 × 8 0.070λ × 0.070λ	2.6, 6.3, 9.3	S, C, X	14.3	Antenna gain enhancement
Proposed	2021	8 × 8, 0.06λ × 0.06λ	2.48, 4.28, 9.36, 13.7	C, S, X, Ku	15.1	Gain enhancement of multiband antenna

## Conclusion

In this article, an ENG metamaterial based on meaner line resonators is presented that shows near-zero permeability and refractive index. This MTM show multiple resonances of transmission coefficient covering S, C, X, and Ku bands. The calculated EMR is 15.1 that satisfies the stringent criteria of MTM, dimension < λ/10. This high EMR also indicates compactness, indicating that this MTM can be implemented with small-sized devices. The equivalent circuit is modeled in ADS, and the response of S_21_ exhibits excellent matching with the simulation result of CST. A comparison of the measured result with simulation shows close similarity. The investigation of the metamaterial phenomena is also accomplished rigorously with the help of surface current, electric and magnetic field analysis. The performance analysis of different arrays exhibits close similarity with the outcomes of the unit cell. Moreover, the frequency hopping characteristics of the proposed MTM has been investigated through numerical simulations by open and short-circuited the split gaps that help to realize the optimization of the proposed MTM’s performance at different resonance frequencies. Selection of appropriate resonance frequencies can be possible by applying short circuits at the split gaps depending on applications. Due to its small dimension, NZI property, high EMR, along with frequency hopping characteristics, the proposed ENG metamaterial can be utilized for multiband microwave applications such as gain enhancement of multiband antennas.
